# C2orf62 and TTC17 Are Involved in Actin Organization and Ciliogenesis in Zebrafish and Human

**DOI:** 10.1371/journal.pone.0086476

**Published:** 2014-01-27

**Authors:** Franck Bontems, Richard J. Fish, Irene Borlat, Frédérique Lembo, Sophie Chocu, Frédéric Chalmel, Jean-Paul Borg, Charles Pineau, Marguerite Neerman-Arbez, Amos Bairoch, Lydie Lane

**Affiliations:** 1 Department of Human Protein Sciences, Faculty of Medicine, University of Geneva, Geneva, Switzerland; 2 Department of Genetic Medicine and Development, Faculty of Medicine, University of Geneva, Geneva, Switzerland; 3 CRCM - Inserm U1068, Marseille, France; 4 Institut Paoli-Calmettes, Marseille, France; 5 CNRS UMR7258, Marseille, France; 6 Aix-Marseille University, Marseille, France; 7 IRSET - Inserm U1085, Rennes, France; 8 SIB-Swiss Institute of Bioinformatics, Geneva, Switzerland; University of Illinois at Chicago, United States of America

## Abstract

Vertebrate genomes contain around 20,000 protein-encoding genes, of which a large fraction is still not associated with specific functions. A major task in future genomics will thus be to assign physiological roles to all open reading frames revealed by genome sequencing. Here we show that C2orf62, a highly conserved protein with little homology to characterized proteins, is strongly expressed in testis in zebrafish and mammals, and in various types of ciliated cells during zebrafish development. By yeast two hybrid and GST pull-down, C2orf62 was shown to interact with TTC17, another uncharacterized protein. Depletion of either C2orf62 or TTC17 in human ciliated cells interferes with actin polymerization and reduces the number of primary cilia without changing their length. Zebrafish embryos injected with morpholinos against C2orf62 or TTC17, or with mRNA coding for the C2orf62 C-terminal part containing a RII dimerization/docking (R2D2) – like domain show morphological defects consistent with imperfect ciliogenesis. We provide here the first evidence for a C2orf62-TTC17 axis that would regulate actin polymerization and ciliogenesis.

## Introduction

Cilia are centriole-derived projections from the cell surface present in all vertebrates, in invertebrates such as *Drosophila* spp. or *Caenorhabditis elegans*, in protists like *Tetrahymena thermophila*, and in the green alga *Chlamydomonas reinhardtii* [1]. They are made of a microtubule cytoskeleton, the axoneme, surrounded by a membrane that contains numerous receptors and ion channels [2]. Classically, motile cilia are distinguished from primary/immotile cilia based on the structure of their axoneme: motile cilia have nine outer microtubule doublets with a central pair of microtubules, whereas primary cilia lack the central doublet. Motile cilia from multi-ciliated epithelial cells mediate fluid flows inside the organism through mechanically coordinated beating. They are responsible for functions such as mucus clearance in the trachea, egg removal in the fallopian tube or cerebrospinal fluid flow in the brain. Motile monocilia that direct the fluid flow in the embryonic node (Kupffer’s vesicle in Zebrafish (*Danio rerio*)) are responsible for the left/right asymmetrical organization of the vertebrate body [3] [4] [5] [6] [7]. Flagella of protozoans or sperm cells are long motile cilia that allow the motility of entire cells.

A vast range of vertebrate cells are able to assemble a single, immotile primary cilium when they exit from the cell cycle. These primary cilia perform important sensing functions during development and in adult homeostasis. In fish, they are found in the neuromasts of the lateral line to sense mechanical changes in water, in the kidney to sense mechanic flow in the renal ducts, and in the brain [8]. Specialized forms of primary cilia are found in sensory cells such as olfactory sensory neurons or retinal cells [9], where they concentrate and organize sensory signaling molecules.

Regardless of its structure, the axoneme is anchored at the cell surface by the basal body, made of nine triplets of microtubules surrounding the cartwheel. The basal body of the primary cilium derives from the mother centriole during specific phases of the cell cycle. The basal bodies of multi-ciliated epithelial cells are produced de novo by centriole multiplication [10]. In both cases, the transfer of centrioles to the apical plasma membrane depends on the actin network [11].

Recent proteomic, genomic and bioinformatic analyses have allowed cataloguing of over 1,000 centrosome-, basal body- or cilia/flagella-associated proteins, collectively referred to as the “ciliome”, that can be explored in specialized databases such as Centrosomedb [12], the Ciliome Database [13], Ciliaproteome [14], and Cildb [15]. Defects in some of these genes cause disorders called ciliopathies [16], which encompass many symptoms including sensory defects, developmental delay, obesity, diabetes, kidney anomalies, skeletal dysplasia, situs invertus, genital and fertility problems. Recently, dozens of additional genes involved in ciliogenesis have been identified using RNA interference on human cells [17] [18]. However, some causative genes in these diseases are still missing. We postulated that candidate genes could be found among the thousands of human proteins that are only predicted from transcriptomic data and still await experimental validation and characterization [19].

Among a set of 5,200 poorly characterized human proteins - according to neXtProt annotation[20], 1,049 were found by BLAST analysis to have a phylogenetic profile compatible with an involvement in ciliogenesis, *i.e.* conserved in vertebrates but not in the following organisms devoid of cilia: *Escherichia coli*, *Bacillus subtilis*, *Methanocaldococcus* sp., *Saccharomyces cerevisiae* and *Dictyostelium discoideum* [1].

The zebrafish model has been extensively used to study ciliogenesis [8] [7] [21]. It allows different approaches compared to mammalian models, including a fast and readily observable development, relatively easy establishment of transgenic reporters and the possibility to modulate protein expression by injection of morpholinos (MOs) at early developmental stages [22]. Because MO strategies are generally more promising for genes that are well conserved and have no functional equivalog, proteins having at least one paralog in human or zebrafish or whose sequences were divergent between zebrafish and human were eliminated from the preliminary set. That led to a final set of 283 poorly characterized proteins with no paralog and a phylogenetic profile compatible with a role in ciliogenesis.

This report presents the first characterization of one of these proteins, C2orf62, in zebrafish embryo and human cell lines. C2orf62 is present in ciliated cells throughout zebrafish embryonic development. In human, it is expressed in ciliated tissues, notably in testis at the moment when sperm cell flagella are formed. C2orf62 was found to interact with TTC17, another uncharacterized protein. We show that C2orf62 and TTC17 are involved in primary ciliogenesis in human cells and modulate actin polymerization. Moreover, down-regulation of C2orf62 or TTC17 by MO in zebrafish embryos or overexpression of C2orf62 C-terminus induces morphological features classically associated with ciliogenesis defects.

## Results

### C2orf62 is a candidate ciliogenesis gene

We reasoned that proteins involved in ciliogenesis would start to be expressed in zebrafish at 10–12 hours post fertilization (hpf), during the formation of the ciliated Kupffer’s vesicle [4] [6] [7]. A RT-PCR screen was performed on 120 genes from the list of candidates established by bioinformatics (see introduction), and 18 matched this expression criterion (R.J.F., unpublished data). We chose to start our validation work with C2orf62 because Switzerland is committed to investigate human chromosome 2 proteins within the HUPO Chromosome-Centric Human Proteome Project, whose aim is to annotate all human proteins [23].

**Figure 1:**
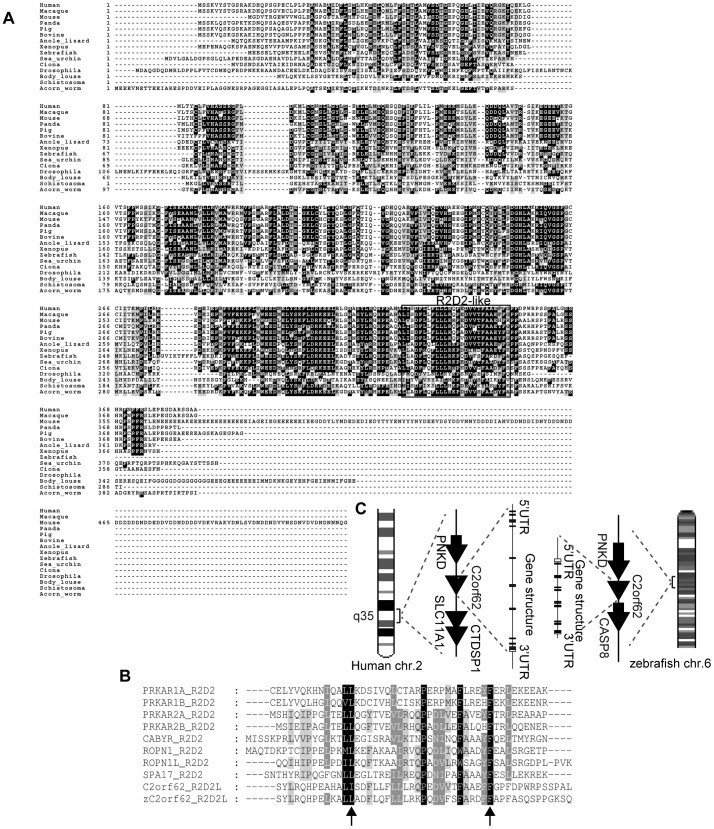
C2orf62 is widely conserved in metazoans. (**A**) Multiple protein sequence alignment of C2orf62 orthologs. Accession numbers of sequences are: **Human**, Q7Z7H3; **Macaque**, F7CRL9; **Mouse**, B9EKE5; **Panda**, XP_002913749.1; **Bovine**, E1BGR4; **Pig**, NP_001177149; **Anole lizard**, XP_003214975; **Xenopus**, Q0IHI3; **Zebrafish**, Q08CH6; **Acorn worm**, XP_002734427.1; **Sea urchin**, XP_793449.3; **Ciona**, XP_002131511.1; **Body louse**, E0VIE2; **Drosophila**, Q9V3B1; **Schistosoma**, G4VER9. Boxed is the R2D2 (PF02197)-like domain (**B**) C2orf62 and zC2orf62 genomic structures and synteny. (**C**) Multiple protein sequence alignment of the R2D2-like domains of human and zebrafish C2orf62, the R2D2 domains of PKA regulatory subunits PRKAR1A, PRKAR1B, PRKAR2A, PRKAR2B and the R2D2 domains of CABYR, ROPN1, ROPN1L and SPA17. The conserved residues that were mutated in zC2orf62 for the functional analysis shown in Fig. S6D are indicated by arrows.

C2orf62 is a 387 amino-acid (aa) protein for which no information concerning function or interacting partners is available in the literature. It is not mentioned in any of the ciliome databases [15] [12]. To our knowledge, it has never been detected by mass spectrometry; its existence has only been validated at transcript level in the brain (BC052750). Using an antibody against aa 217–305 (HPA044818), the Human Protein Atlas [24] team reported an enrichment in heart myocytes and bone marrow cells, but with uncertain reliability. The COSMIC database [25] reports three somatic mutations (R133W, I168V and S162Y) associated with rectal adenocarcinoma samples. Whereas C2orf62 has no annotated functional domain, Pfam [26] analysis shows that the aa 327–352 region is just below the threshold of detection by Pfam of the docking and dimerization domain of protein kinase A II-alpha (R2D2, PF02197) (Fig. 1A). This domain is found at the N-terminus of regulatory subunits of protein kinase A and at the N-terminus of four AKAP-binding proteins found in ciliated cells and highly expressed in testes, where it provides the dimerization interface and the binding site for A-kinase-anchoring proteins (AKAPs) [27] [28] [29] (Fig. 1B).

C2orf62 orthologs are confined to Metazoa: no ortholog was found by TBLASTN in plants, Fungi, Amoebozoa, Alveolata, or Stramenopiles. C2orf62 is well-conserved in Chordates (all vertebrates and Ciona), Echinoderms (Sea urchin), Hemichordata (Acorn worm), Insecta (Drosophila, Body louse) and Plathelminthes (Schistosoma) (Fig. 1A). The sequences of zebrafish and human proteins share 36% identity. Gene synteny is partially conserved, with PNKD as upstream adjacent gene in both organisms (Fig. 1C).

According to UniGene, the mouse ortholog Gm216 is specifically expressed in brain, testis and nasopharynx (www.ncbi.nlm.nih.gov/UniGene/clust.cgi?ORG=Mm&CID=310460). The Drosophila ortholog CG13243 is specifically expressed in adult testis (flybase.org/reports/FBgn0028903.html), and the protein has been identified in sperm [30]. The high conservation level of C2orf62 across metazoans and its restricted expression in mouse and Drosophila ciliated tissues prompted us to analyze its expression profile in zebrafish.

### zC2orf62 is expressed in ciliated cells during embryonic development

**Figure 2:**
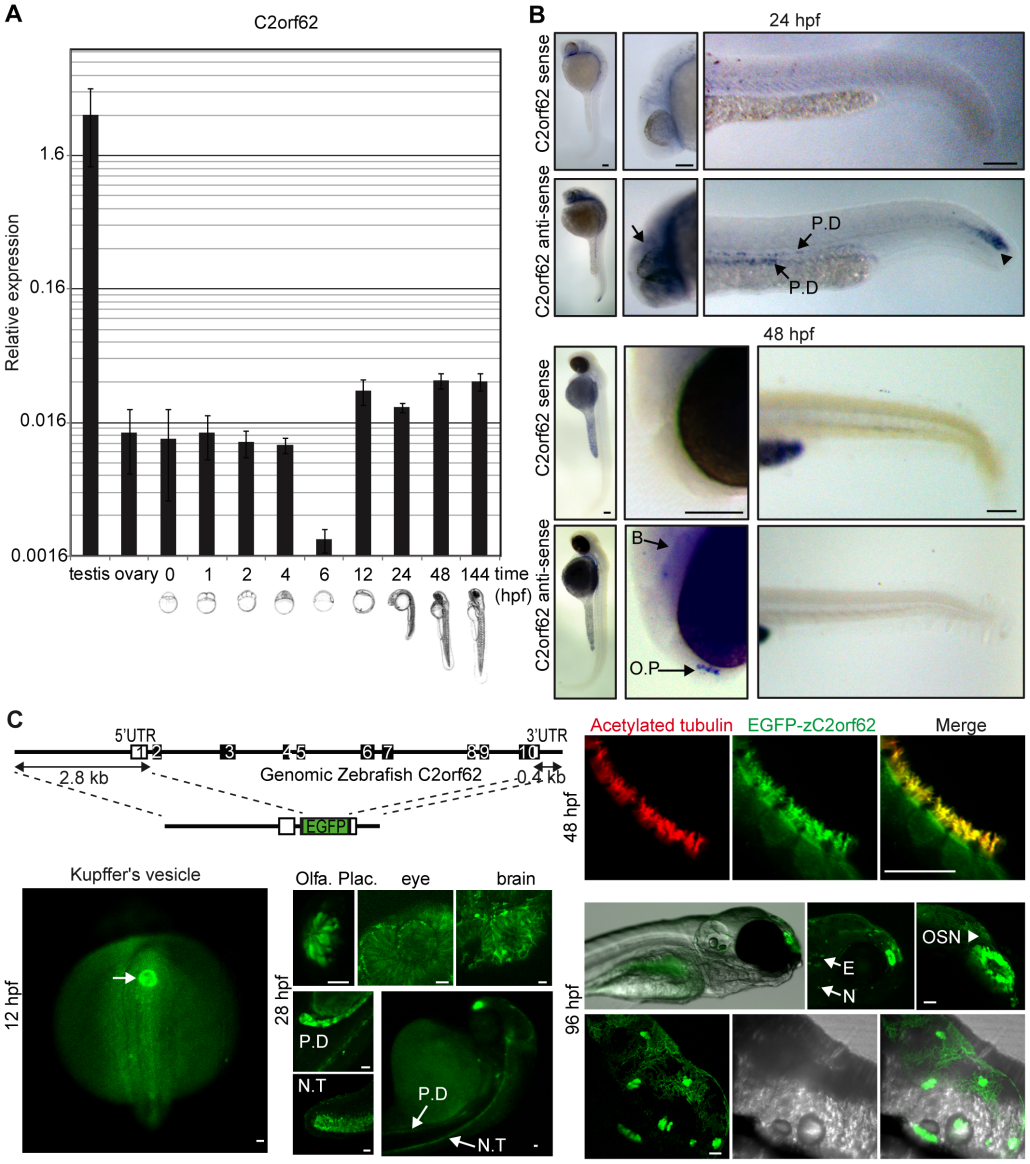
zC2orf62 is expressed in ciliated structures during embryonic development and highly expressed in adult testis. (**A**) zC2orf62 mRNA levels measured by RT-qPCR at various developmental stages. zC2orf62 is highly expressed in adult testis and during almost all embryonic development. It is down-regulated at shield stage (6 hpf) and re-expressed at tail bud (12 hpf) when Kupffer's vesicle forms. (**B**) Whole mount in situ hybridization of zC2orf62 mRNA at 24 hpf and 48 hpf. At 24 hpf, zC2orf62 mRNA is detected at the end of neural tube formation on the tail extremity (arrowhead), in pronephric ducts (P.D.) and in the brain (arrows). At 48 hpf, zC2orf62 is expressed in forebrain (B) and olfactory pits (O.P.) (arrows). Scale bars, 100 µm. (**C**) A construct containing zC2orf62 5′-UTR, 3′-UTR and potential regulatory sequences in which zC2orf62 coding sequence was replaced by EGFP was generated and inserted randomly inside the zebrafish genome. Resulting transgenic EGFP-zC2orf62 reporter fish were observed at 12 hpf using a fluorescent stereomicroscope and at 28, 48 and 96 hpf using a confocal microscope. EGFP is expressed at 12 hpf in Kupffer's vesicle, and at 28 hpf in olfactory placode, eyes, brain, neural tube (N.T), and pronephric ducts (P.D). At 48 hpf, EGFP is expressed in ciliated cells of the olfactory organ (positive for α-acetylated tubulin (red)). At 96 hpf, EGFP is also expressed in neuromast cells (N), olfactory sensory neurons (OSN) and in the ears (E and lower enlarged panel). Scale bars, 25 µm. See also Figs S1, S2.

In the absence of expression data for zC2orf62 (zgc:153063) in the Zfin database [31], we sought to confirm our initial RT-PCR results by quantitative RT-PCR (RT-qPCR) (Figs 2A, S1). As expected, zC2orf62 mRNA is not detectable at 6 hpf and clearly expressed after 12 hpf, in a development timing that corresponds to optic vesicle and Kupffer’s vesicle development and neural keel formation [32] [3] [4]. zC2orf62 is also expressed between 0 and 4 hpf during maternal and early zygotic periods [33], and strongly in adult testis (about 240 times more than in ovary and embryo).

zC2orf62 expression pattern was investigated by in situ hybridization at 24 and 48 hpf (Fig. 2B). At 24 hpf, zC2orf62 is expressed in the extremity of neural tube formation inside the tail, in brain and in pronephric ducts (future kidney) (Fig. 2B top). At 48 hpf, zC2orf62 expression is restricted to brain and olfactory pits. No expression in pronephric ducts and neural tube can be observed (Fig. 2B down). To further characterize zC2orf62 expression during development, reporter transgenic fish lines expressing EGFP under the control of zC2orf62 promoter were established (Fig. 2C). Fluorescence starts to be observed around 12 hpf in Kupffer’s vesicle. At 28 hpf, fluorescence was visualized in brain, neural tube and pronephric ducts, consistent with in situ results, as well as in the olfactory placode and the eye (Fig. 2C). At 48 hpf, expression is detected in the ciliated cells of the olfactory placode, ear, neuromasts and pronephric ducts, visualized by acetyl-tubulin labeling (Figs 2C, S2A). At 96 hpf, fluorescence was observed in neuromasts, olfactory sensory neurons, and in the ear. Within the ears, the EGFP signal was restricted to sensory patches (anterior and posterior maculae, anterior, lateral and posterior cristae), which contain ciliated hair cells [34] (Figs 2C, S2B).

Taken together, expression profiles obtained with transgenic reporter fish and by in situ hybridization are well correlated and show early zC2orf62 expression in Kupffer vesicle, neural tube, pronephric ducts and brain, followed by expression in sensory structures of the olfactory placode, eye, ear and neuromasts.

Unfortunately, our antibody against human C2orf62 does not cross-react with zC2orf62, preventing us from confirming the expression profile of zC2orf62 at protein level, and defining its subcellular location in ciliated cells. Therefore, C2orf62 expression was analyzed in mammalian tissues.

### C2orf62 is expressed in mammalian germ cells and ciliated cells

**Figure 3:**
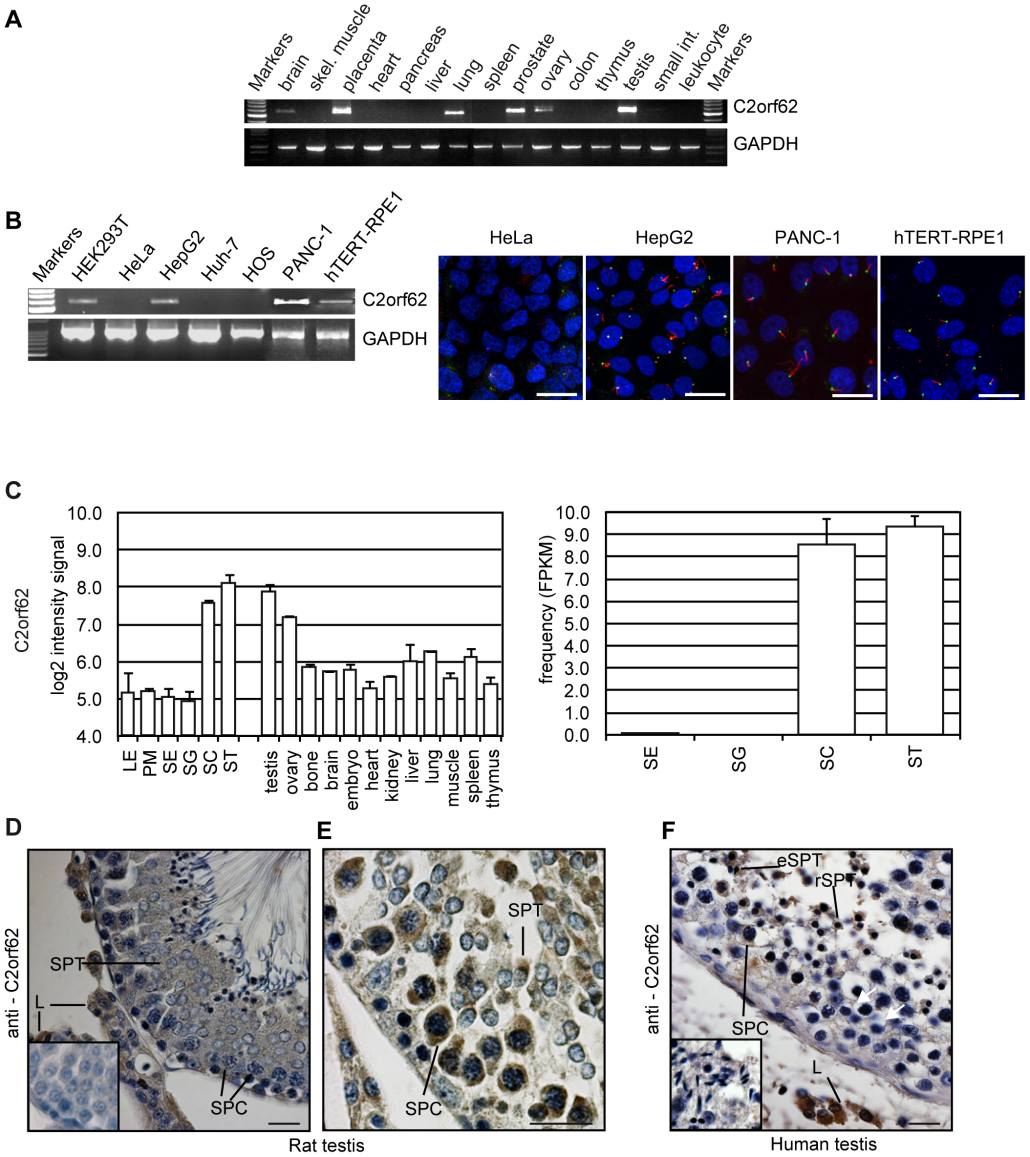
Mammalian C2orf62 is expressed in ciliated tissues and cell lines. (**A**) PCR performed on a pre-normalized tissue cDNA panel (Clontech) showing that C2orf62 is highly expressed in testis, placenta, prostate and lung, and moderately in ovary and brain. GAPDH was assessed in parallel for comparison. (**B**) **Left panel**: RT-PCR showing C2orf62 expression in HEK293T, HepG2, PANC-1 and hTERT-RPE1 but not in HeLa, Huh-7 and HOS cells. **Right panel**: HeLa, HepG2, PANC-1 and hTERT-RPE1 cells were serum-starved for 48 hours (hTERT-RPE1 cells) or 72 hours (the 3 other cell lines). Cilia were visualized by immunofluorescence using anti α-acetylated tubulin (red), centrioles by immunofluorescence using anti pericentrin (green) and nuclei by DAPI coloration (blue). In contrast to HepG2, PANC-1 and hTERT-RPE1 cells, HeLa cells were unable to grow cilia. Scale bars, 25 µm. (**C**) Histograms showing that C2orf62 transcript expression levels based on Affymetrix Rat Exon 1.0 ST (left) and Illumina RNA-seq (right) are higher in rat testis and ovary than in other tissues, and higher in spermatocytes (SC) and spermatids (ST) than in somatic testicular cells [Leydig (LE), peritubular myoid (PM) and Sertoli (SE)] and in spermotogonia (SG). (**D,E**) Transverse sections of a testis from an adult rat showing C2orf62 immunoreactivity (**D**) in the cytoplasm of pachytene spermatocytes (SPC) and of early and elongating spermatids (SPT) at stage VII of spermatogenesis (with strong staining in Leydig cells (L)), and (**E**) at higher magnification, in the cytoplasm of pachytene spermatocytes (SPC) and early spermatids (SPT), here at stage IX of spermatogenesis. (**F**) Transverse section of a human testis showing C2orf62 immunoreactivity in the cytoplasm of pachytene spermatocytes (SPC), early/round (rSPT) and elongating spermatids (eSPT). A strong staining in the cytoplasm of Leydig cells (L) is also visible. Inserts: negative controls with preimmune serum. Scale bars, 50 µm.

By RT-PCR, we showed that C2orf62 is highly expressed in human testis, placenta, prostate and lung, moderately in ovary and brain, and undetectable in other tissues (Fig. 3A). Genome-wide expression profiling in rat (manuscript in preparation, Chalmel et al.) shows a stronger expression in testis and ovary than in other tissues (Fig. 3C). In rat testis, C2orf62 is highly expressed in pachytene spermatocytes and round spermatids compared to spermatogonia and somatic cells (Fig. 3C), which indicates a specific expression in meiotic and post-meiotic germ cells.

Immunohistochemistry on rat testis shows that C2orf62 protein is enriched in the cytoplasm of spermatocytes at the pachytene stage and concentrated in elongating spermatids (Fig. 3D main section-stage VII). Signal is also visible in the cytoplasm of elongated spermatids (Fig. 3E–stage IX), but there is no accumulation in late spermatids prior to their release into the lumen (stage VII). Signal is absent in Sertoli cells, but unexpectedly present in Leydig cells (Fig. 3D). A similar localization was found in human testis with a high staining in the cytoplasm of round and elongating spermatids and a lower one in pachytene spermatocytes. An intense staining was also visible in the cytoplasm of Leydig cells (Fig. 3F).

C2orf62 is expressed in the human cilia-forming cell lines HEK293T [35], PANC-1 [36] and hTERT-RPE1 [37] but not in HeLa [38], Huh-7 and HOS cell lines devoid of cilia (Fig. 3B). C2orf62 mRNA was also detected in HepG2 cells. Hepatocytes are classically considered to be devoid of cilia, but some cancerous cell lines derived from hepatocytes do form cilia [39]. We verified that, in our hands, HepG2 cells formed cilia upon serum starvation, like PANC-1 and hTERT-RPE1 cells (Fig. 3B).

**Figure 4:**
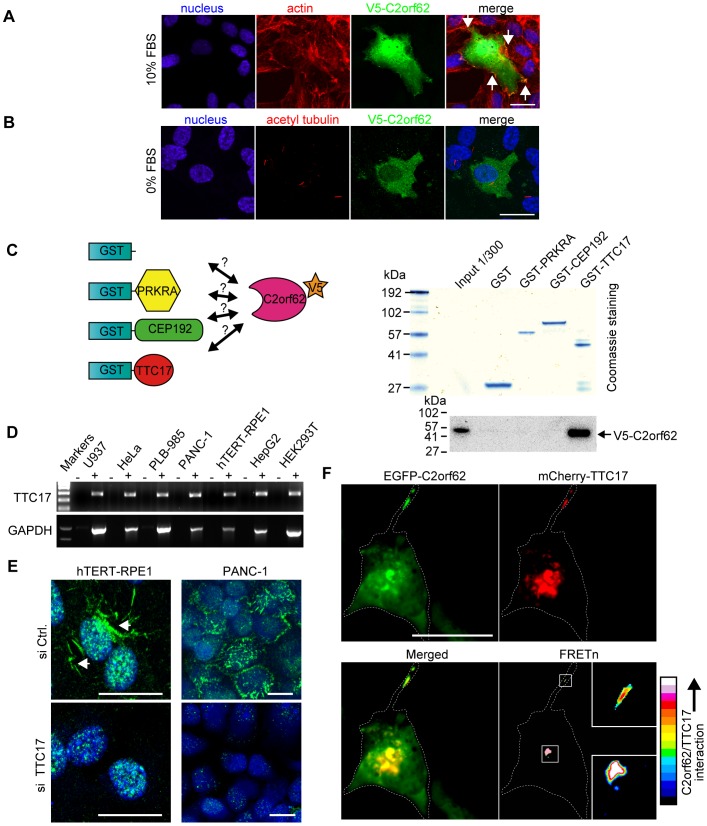
C2orf62 interacts with TTC17 in mammalian cells. (**A**) When overexpressed in hTERT-RPE1 cells grown at 10% FBS, V5-C2orf62 localizes in the cytoplasm, nucleus and in F-actin-rich zones of the plasma membrane (arrows). See also EGFP-C2orf62 live cell imaging in movie S1. (**B**) Ciliogenesis was induced by serum starvation in hTERT-RPE1 cells transfected with V5-C2orf62. V5-C2orf62 (green) was not detected in cilia visualized using anti α-acetylated tubulin (red). Scale bars, 25 µm. (**C**) Beads coated with GST, PRKRA-GST, CEP192-GST and TTC17-GST were used for pull-down experiments on V5-C2orf62-transfected HEK293T cell lysates. V5-C2orf62 only bound to TTC17-GST beads. See also Table S1. (**D**) RT-PCR showing TTC17 expression in every tested cell line (U937, HeLa, PLB-985, PANC-1, hTERT-RPE1, HepG2 and HEK293T). GAPDH was assessed in parallel for comparison. (**E**) Subcellular localization of endogenous TTC17 was studied by immunofluorescence on hTERT-RPE1 and PANC-1 cells transfected either with a control siRNA (si Ctrl) or with siRNA against TTC17 (si TTC17). Despite a strong background staining in the nucleus, a specific staining that disappeared with si TTC17 treatment could be seen in hTERT-RPE1 cells (see also Fig. S3). The staining was more intense, with less background, in PANC-1 cells. Scale bars, 25 µm. (**F**) hTERT-RPE1 cells were co-transfected with EGFP-C2orf62 and mCherry-TTC17 (red) and observed 24h later by confocal microscopy. Both fusion proteins were enriched in the same areas, including cell surface protrusions. The pseudocolor images represent FRET signals corrected for bleed-through using the normalized FRET method (FRETn), with white and red indicating higher interaction levels. Colocalisation was detectable by FRETn, suggesting a stable interaction. Scale bars, 25 µm.

Although our antibody against human C2orf62 works well in tissues like testis, which express C2orf62 strongly, we failed to detect a specific signal in hTERT-RPE1 or PANC-1 cell lines (data not shown). Therefore, a C2orf62 overexpression strategy was chosen to precise C2orf62 subcellular location in ciliated hTERT-RPE1 cells. V5-C2orf62 shows variable localization in the cytoplasm, nucleus and F-actin rich zones of the plasma membrane, but is always excluded from cilia, as shown by the lack of co-localization with acetylated tubulin (Fig. 4A,B). Same results were obtained in PANC-1 cells (data not shown).

The variability in the observed V5-C2orf62 localization in fixed cells might be explained by a dynamic localization cycle of the protein. To assess this point, overexpressed EGFP-C2orf62 was observed by time lapse microscopy. EGFP-C2orf62 was observed in the whole cell but concentrated in dynamic plasma membrane protrusions that appear and disappear in less than 5 min (Movie S1).

### C2orf62 interacts with TTC17 (Tetratricopeptide repeat protein 17)

Since C2orf62 is expressed in brain (Fig. 3A) and potentially involved in development, we screened a human fetal brain cDNA library by yeast two-hybrid using full-length C2orf62 as bait, in order to identify possible partners. Among the 10 interacting protein-coding clones (Table S1), 3 were selected as relevant: PRKRA, which plays a role in ciliogenesis [18], CEP192, which is involved in cell cycle and cilia formation [40] [41] and TTC17 because some proteins containing tetratricopeptide repeats are involved in intraflagellar transport or cilia formation [42] [43] [44]. The full-length sequence of PRKRA and the sequences of the C2orf62-interacting regions of CEP192 (aa 1501–1941) and TTC17 (aa 945–1041) were produced as GST fusion proteins and used to precipitate V5-C2orf62 from a HEK293T cell lysate. Only TTC17 interacted with C2orf62 under these conditions (Fig. 4C).

Since TTC17 has not been characterized yet, we analysed its expression in mammals in order to investigate whether this interaction could be relevant in ciliated cells.

Ttc17 is ubiquitously expressed in rat tissues. Within rat testes, Ttc17 mRNA is detectable in germ cells as well as in somatic cells (Fig. S3A). TTC17 is also expressed in every tested human cell line (Fig. 4D). According to the Human Protein Atlas (HPA) [24], TTC17 protein is mainly located in respiratory epithelium, fallopian tube and epididymis, and localizes to both the cytosol and the plasma membrane in different cell lines (HPA038508). Using the same antibody, we found that TTC17 distribution in mammalian testis closely matches the distribution of C2orf62, being undetectable in Sertoli cells, present in germ cells and enriched in spermatocytes, both in rat and in human (Fig. S3B).

Although the HPA038508 antibody gave a strong nuclear background signal, a specific filamentous staining that was abolished by siRNA against TTC17 could be observed in hTERT-RPE1 cells (Fig. 4E). The staining was more intense in PANC-1 cells and completely abolished by siRNA. The observed localization (Fig. 4E) is similar to what was reported by HPA in A-431 and U-251 MG cells. Interestingly, the signal detected in hTERT-RPE1 cells was strongly enhanced in mitotic cells, from metaphase to telophase (Fig. S3C).

TTC17 and C2orf62 were co-expressed in hTERT-RPE-1 cells as mCherry and EGFP fusion proteins, respectively, and observed by confocal microscopy. As previously described using EGFP-C2orf62 alone (Movie S1), both proteins were found to localize in discrete parts of the cytoplasm and in cell protrusions (Fig. 4F). Colocalization was confirmed by FRET analysis (Fig. 4F), which indicates that C2orf62 and TTC17 may interact in ciliated cells.

### C2orf62 and TTC17 are involved in ciliogenesis in human cells

**Figure 5:**
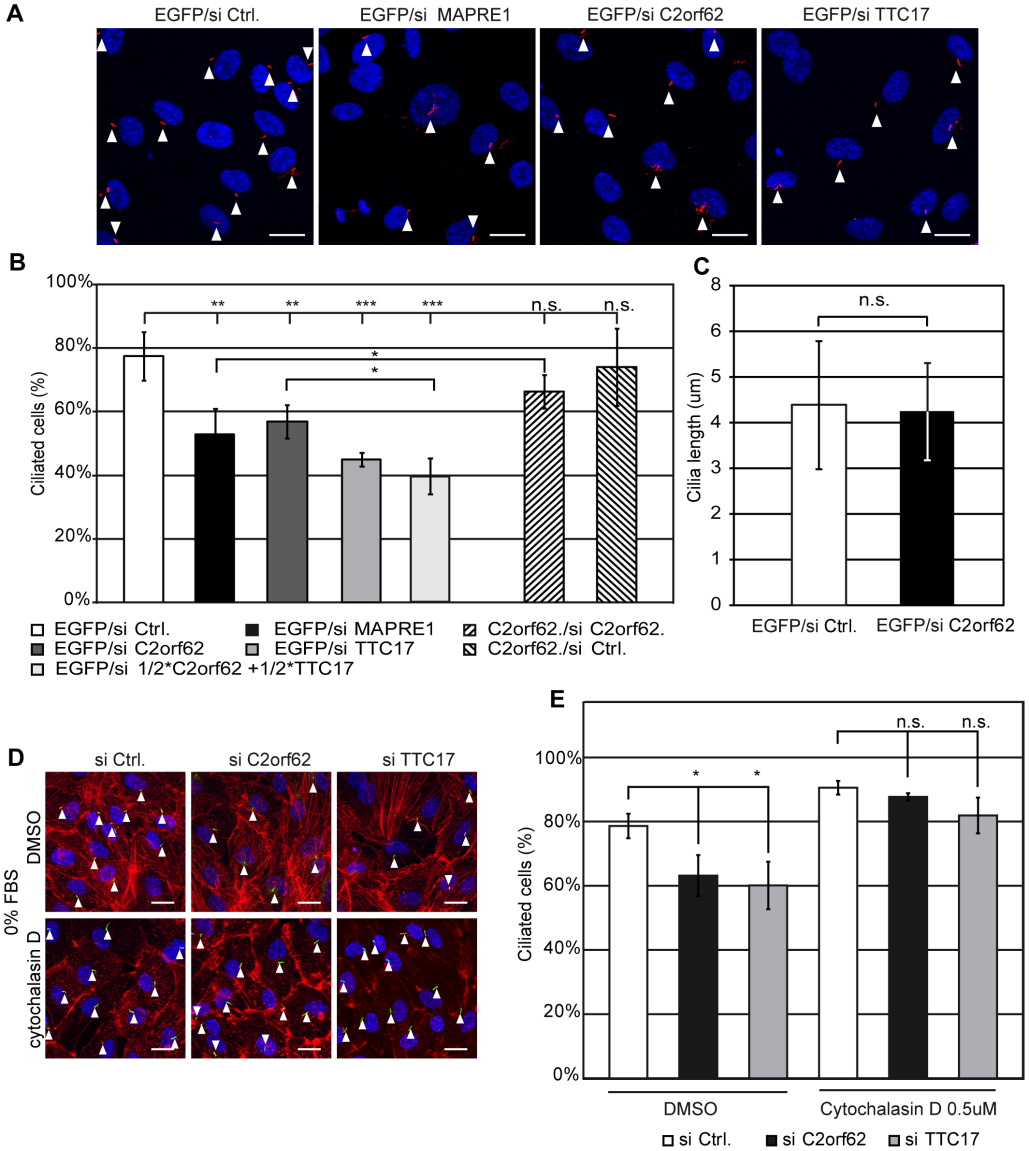
C2orf62 and TTC17 act on ciliogenesis by modulating actin polymerization. Ciliogenesis was induced by serum starvation in hTERT-RPE1 cells transfected with combinations of siRNA and plasmids as indicated **(A-C)** Cells transiently expressing EGFP or C2orf62 were transfected with siRNA against C2orf62, MAPRE1 or TTC17 (50 µM), with both siRNA against C2orf62 and TTC17 (25 µM each), or with a control siRNA. Cilia were visualized by immunofluorescence using anti α-acetylated tubulin (red) and nuclei by DAPI (blue). (**A**) Cells transfected with siRNA against MAPRE1, C2orf62 or TTC17 have less cilia than cells transfected with control siRNA. Scale bars, 25 µm. (**B**) The bar graph shows the percentages of ciliated cells obtained in a minimum of 3 independent experiments with 100 cells counted per condition in each experiment. C2orf62, TTC17 and MAPRE1 siRNA significantly reduce cilia numbers, and effects of C2orf62 and TTC17 siRNA are synergic. Effect of C2orf62 siRNA can be rescued by C2orf62 overexpression (C2orf62 siRNA/Ctrl siRNA**P = 0.0026; MAPRE1 siRNA /Ctrl siRNA **P = 0.0034; TTC17 siRNA/Ctrl siRNA ***P = 0.0005; ½C2orf62 + ½TTC17 siRNA/Ctrl siRNA ***P = 0.0004; C2orf62 + C2orf62 siRNA/EGFP + C2orf62 siRNA *P = 0.035; ½C2orf62 + ½TTC17 siRNA/C2orf62 siRNA *P = 0.0127). **(C)** C2orf62 siRNA does not affect cilia length. (**D, E**) Cells transfected with 50 µM siRNA against C2orf62 or TTC17 or with a control siRNA were serum-starved in the presence or absence of 500 nM cytochalasin D. Cilia were visualized by immunofluorescence using anti-acetylated α-tubulin antibody (green), F-actin by phalloidin (red) and nuclei by DAPI (blue). Scale bars, 25 µm. (**D**) Both F-actin polymerization and the reduction in cilia numbers induced by siRNA are reversed by cytochalasin D. (**E**) The bar graph shows the percentages of ciliated cells obtained in each condition. siRNA against C2orf62 or TTC17 significantly reduce the number of cilia in DMSO-treated cells (C2orf62 siRNA/Ctrl siRNA *P = 0.032; TTC17 siRNA/Ctrl siRNA *P = 0.031). Cells treated with Cytochalasin D exhibit more cilia than cells treated with vehicle (DMSO) in all conditions (C2orf62 siRNA/C2orf62 siRNA + CytD *P = 0.018; C2orf62 siRNA/C2orf62 siRNA + CytD *P = 0.019; Ctrl siRNA/Ctrl siRNA + CytD P = 0–016). In the presence of Cytochalasin D, siRNA against C2orf62 or TTC17 do not affect the number of cilia. See also effects of C2orf62 siRNA on PANC-1 cells in Fig. S4B.

The possible implication of C2orf62 and TTC17 in ciliogenesis was investigated by RNA interference in human ciliated cells. Ciliogenesis was induced by serum starvation 24 h after transfection with control siRNA or with siRNA against C2orf62, TTC17 or MAPRE1/EB1, a microtubule (MT) plus-end-tracking protein involved in several microtubule-dependent cellular processes, including primary cilia assembly [45], mitosis and cell migration [46]. siRNA against C2orf62, TTC17 or MAPRE1/EB1 reduced the number of ciliated cells obtained after serum starvation, both in hTERT-RPE1 cells (Fig. 5A,B) and in PANC-1 cells (data not shown). The combination of siRNA against C2orf62 and TTC17 has a stronger effect than a double dose of siRNA against C2orf62 (Fig. 5B), which suggests that C2orf62 and TTC17 may contribute to the same biological function.

Ciliogenesis in hTERT-RPE1 cells can only be induced by serum deprivation if cells are spatially confined [47]. To test if the observed effects of siRNA on ciliogenesis could be indirectly due to an decrease in cell density, we counted cells 48 h and 72 h after siRNA transfection. siRNA against TTC17 had no effect at either time point. siRNA against C2orf62 had no effect at 48 h and slightly reduced cell numbers to 80% of controls 72 h after transfection, as did siRNA against MAPRE1/EB1 (Fig. S4A). Similar effects on cell growth were previously described on COLO 320 cells using siRNA against MAPRE1/EB1 [46]. In our ciliogenesis experiments, serum starvation was performed 24 hours after siRNA transfection. At this time point, none of the siRNA had an effect on cell density, excluding the possibility that the observed defect of ciliogenesis would indirectly result from a decreased density of cells at the time of serum starvation. Furthermore, the length of cilia in cells treated with siRNA against C2orf62 was similar to the length of cilia in control cells (4.4 um +/– 1.4 versus 4.25 um +/– 1.1) (Fig. 5C), and similar to the reported length of cilia when starvation is done on confined hTERT-RPE1 cells (5 um) [47]. The lack of effect on siRNA against C2orf62 on cilia length suggests that C2orf62 would be involved in cilia initiation rather than in cilia elongation.

### C2orf62 and TTC17 modulate actin polymerization in ciliated human cells

Since actin dynamics have been shown to affect ciliogenesis [17], we tested whether actin structures could mediate some of the observed effects of siRNA against C2orf62 and TTC17 on primary cilium growth. hTERT-RPE1 cells with down-regulated C2orf62 or TTC17 were labeled for F-actin and alpha-acetylated tubulin after serum starvation. The reduction in cilia number induced by siRNA against TTC17 or C2orf62 was accompanied by extensive actin polymerization (Fig. 5D). Similar effects were observed using siRNA against C2orf62 on PANC-1 cells (Fig. S4B). Interestingly, siRNA against C2orf62 also induced moderate actin polymerization on unstarved cells (Fig. S4B). These observations suggest that siRNA against C2orf62 and TTC17 may prevent cilia initiation by modifying actin architecture.

Cytochalasin D is an F-actin destabilizer that enhances ciliogenesis at submicromolar concentrations [17] [48]. We verified that cytochalasin D (500 nM) increases ciliogenesis in hTERT-RPE1 cells, as previously described [47]. Whereas siRNA against TTC17 or C2orf62 inhibited ciliogenesis in DMSO-treated hTERT-RPE1 cells, they were without effect in presence of cytochalasin D (Figs 5D,E), confirming that C2orf62 and TTC17 may act on ciliogenesis by interfering with F-actin dynamics.

### zC2orf62 and zTTC17 knock-down induce ciliogenesis defects in vivo

In order to confirm the role of C2orf62 and TTC17 in ciliogenesis in vivo, a MO strategy carefully designed to avoid off-target effects [22] was employed. Two MOs were designed for each gene. C2orf62_1 MO targets C2orf62 start codon, C2orf62_2 MO targets the splice site between intron 2 and exon 3 of zC2orf62 pre-mRNA, TTC17_1 MO targets the splice site between exon 1 and intron 1 of zTTC17 pre-mRNA, TTC17_2 MO targets the splice site between exon 2 and intron 2 of zTTC17 pre-mRNA. At 5 ng, C2orf62_1 MO abolished the fluorescence in embryos injected with zC2orf62-EGFP mRNA (Fig. S5D), demonstrating its efficiency. The inhibitory effect of C2orf62_2 MO on zC2orf62 splicing was checked by RT-PCR on embryos injected with 5 ng C2orf62_2 MO (Fig. S5E).

**Figure 6:**
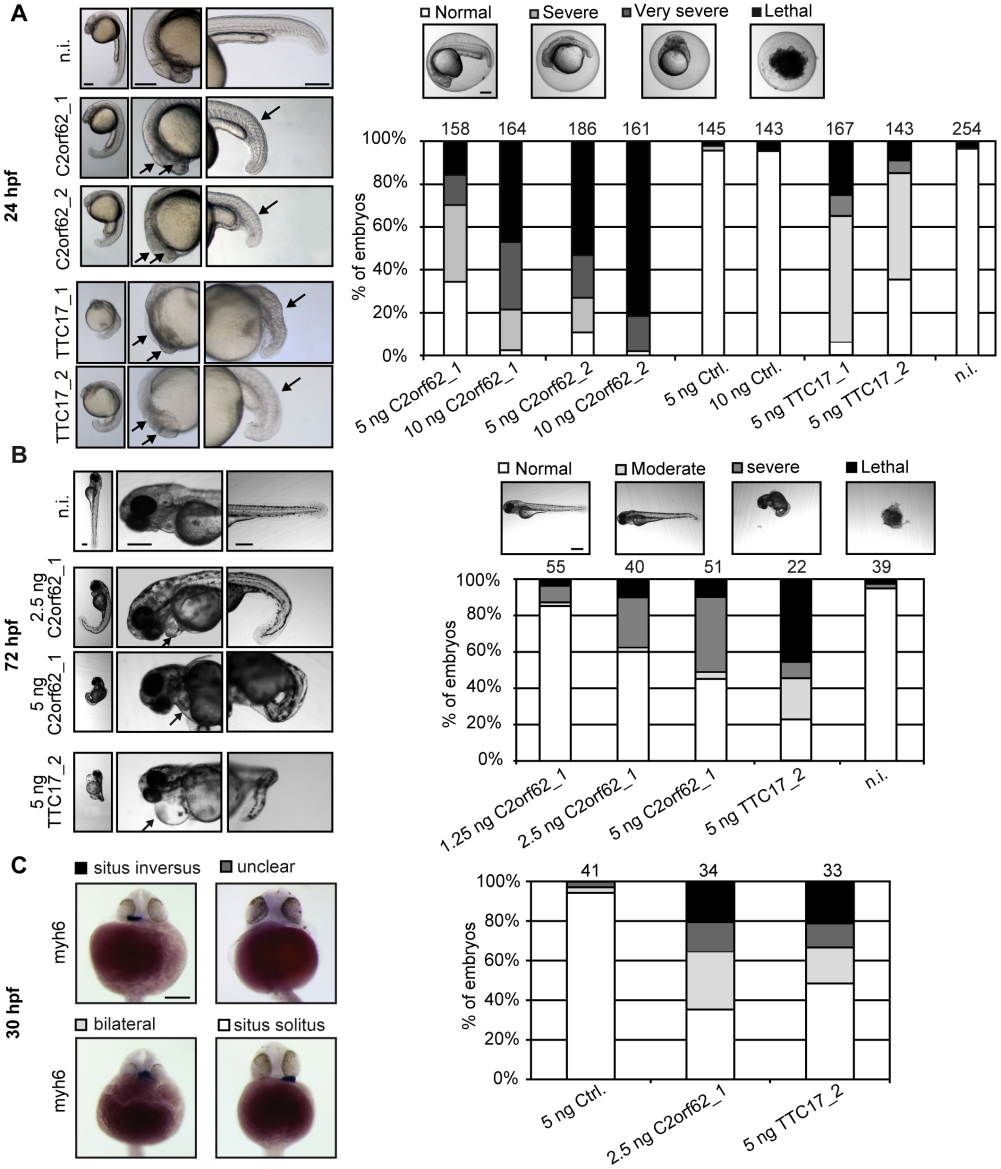
Knockdown of zC2orf62 or zTTC17 in embryos results in body curvature, head defects, and heart positioning defects. Zebrafish embryos were either not injected (n.i.) or injected with different quantities of Ctrl. MO, C2orf62_1 MO, C2orf62_2 MO, TTC17_1 MO or TTC17_2 MO and observed under a stereomicroscope. Scale bars, 200 µm. (**A**) Embryos injected with 5 ng C2orf62_1 MO, C2orf62_2 MO, TTC17_1 MO or TTC17_2 MO display the same phenotype at 24 hpf characterized by a curved body, a lack of defined brain structures or necrosis in the developing brain, and eye formation defects (arrows). Phenotypic traits were classified into four categories, each being associated with one grey code, as reported in the bar graph: normal (white, indistinguishable from controls), severe (clear grey, defect of brain structure formation and short body axis), very severe (dark grey, indistinguishable left/right or front/back polarity), and lethal (black). The bar graph shows the percentages of embryos in each category. Injection of 10 ng C2orf62_1 or C2orf62_2 MO or of 5 ng TTC17_1 MO results in >97% severe-to-lethal phenotypes. Injection of 5 ng C2orf62_1 or TTC17_2 MO results in about 65% severe-to-lethal phenotypes. See also the synergy between C2orf62_1 MO and C2orf62_2 MO in fig. S5. (**B**) Embryos were either not injected (n.i.), or injected with 1.25 to 5 ng C2orf62_1 MO or with 5 ng TTC17_2 MO, and observed at 72 hpf. Embryos injected with 5 ng C2orf62_1 or TTC17_2 MO display a curved body, incorrectly defined brain structures and heart edema (arrows). Injected embryos were classified into four categories, each being associated with one grey code, as reported in the bar graph: normal (white, indistinguishable from controls), moderate (clear grey, little body curvature and small heart edema), severe (dark grey, strong body curvature, brain defect morphology and edema), and lethal (black). The bar graph shows the percentages of embryos in each category. Injection of 5 ng C2orf62_1 or TTC17_2 MO results in about 50% severe-to-lethal phenotypes. (**C**) Embryos were labeled for *myh6* at 30 hpf and classified into four categories according to the position of their heart (ventral views, scale bars, 200 µm): situs solitus (white), situs inversus (black), bilateral/midline positioning (light grey) and unclear/undeterminable positioning (dark grey). The bar graph shows the percentage of embryos in each category. About 20% of C2orf62_1 or TTC17_2 MO-injected embryos display situs inversus. See Figs S5 and S6 for further characterization of C2orf62_1 and C2orf62_2 MO.

At 24 hpf, injection of C2orf62_1 MO, C2orf62_2 MO, TTC17_1 MO and TTC17_2 MO resulted in similar phenotypic traits with various severity degrees (Fig. 6A), from minor developmental delay to loss of polarity. These traits include a curved body, a lack of defined brain structures or necrosis in the developing brain, and eye formation defects. The severity of the phenotypic traits was dose-dependent (Fig. 6A and data not shown). Simultaneous injection of C2orf62_1 MO and C2orf62_2 MO at low doses (1.25 ng) gave a synergistic effect (Fig. S5A). MO may provoke off-target effects by inducing the p53-dependent cell death program, which can be avoided by p53 co-knockdown [49]. Co-injection of p53 MO did not change the phenotypic traits induced by C2orf62_2 MO, apart from a slightly reduced brain necrosis (Fig. S5B).

Importantly, the phenotype induced by 10 ng C2orf62_2 MO could be partially alleviated by co-injection of 2 ng zC2orf62 mRNA (Fig. S5C). However, zC2orf62 mRNA itself induced a severe phenotype in 14.5% of embryos (Fig. S5C), which prevented us to test if higher doses could rescue the C2orf62_2 MO-induced phenotypic traits totally. Although it was designed to target zC2orf62 splicing and not its coding sequence, C2orf62_2 MO decreased the fluorescence in embryos injected with zC2orf62-EGFP mRNA (Fig. S5D). This unanticipated inhibitory effect of C2orf62_2 MO on zC2orf62 mRNA translation may explain the modest rescue effect of zC2orf62 mRNA.

To check whether the observed phenotypes at 24 hpf were specific developmental defects and not a global delay in fish development, C2orf62_1 MO-injected embryos were also observed at 48 hpf (Fig. S6B) and 72 hpf (Fig. 6B). Like at 24 hpf, the severity of the phenotypes was dose-dependent, from curly-tipped tail, small cardiac edema and head defects, to strong curvature of the whole body, bigger heart edema, and brain necrosis (Fig. 6B, Fig. S6B). We also verified that the phenotypes induced by injection of TTC17_2 MO at 72 hpf was similar to the phenotypes induced by injection of C2orf62_1 MO. Indeed, injection of either MO at 5 ng resulted in about 50% of severe-to-lethal phenotypes at 72 h (Fig. 6B).

To complete the characterization of C2orf62 and TTC17 MO effects, in situ hybridization experiments using *myosin heavy chain 6* (*myh6*) as heart marker [50] were performed at 30 hpf on embryos injected with 2.5 ng C2orf62_1 MO, 5 ng TTC17_2 MO or 5 ng Ctrl. MO. 65% of C2orf62_1 MO-injected embryos and 52% of TTC17_2 injected embryos have heart positioning defects, and 20% of embryos injected with either C2orf62_1 or TTC17_2 display situs inversus, which is a rare event (<1%) in controls (Fig. 6C).

Additional in situ hybridization experiments using *no tail* (*ntla*) and *sonic hedgehog* (*shha*) as markers for the notochord and floorplate [4] [6] [51] [5] were performed at 24 hpf on embryos, either uninjected (n.i.), or injected with 2.5 ng C2orf62_1 MO or Ctrl. MO. More than 92% of C2orf62_1 MO injected embryos display an undulation of the notochord and floorplate at 24 hpf (Fig. S6A).

Reduced axis length, decreased head size, curved-down body shape, heart position defects and a curled posterior tail were reported at 28–30 hpf in embryos injected with MOs against Cordon-bleu, a protein involved in the development of motile cilia [52]. Abnormal body curvature and heart edema were reported at 48 hpf as consequences of the downregulation of nephrocystin-4, a protein involved in ciliogenesis [53]. Cardiac edema and notochord and tail defects were observed at 72 hpf upon downregulation of meckelin, also required for ciliogenesis [54]. The phenotypes induced by zC2orf62 and TTC17 downregulation at 24–72 hpf are similar to those phenotypes associated with ciliogenesis impairment, suggesting a functional involvement of zC2orf62 and zTTC17 in ciliogenesis in vivo.

In order to directly assess the effect of zC2orf62 knock-down on ciliogenesis, ciliated cells within the olfactory organ were evidenced by α-acetylated tubulin staining. At 48 hpf, embryos injected with 2.5 ng C2orf62_1 MO clearly show a reduction in the number of ciliated cells within the olfactory organ (Fig. S6C).

Altogether, these data show that zC2orf62 and zTTC17 knockdown induce ciliogenesis defects in vivo, confirming the results obtained on human cell lines.

### zC2orf62 C-terminal overexpression induces ciliogenesis defects in vivo

Although C2orf62 has no annotated functional domain, the aa 327–352 region is just below the threshold of detection by Pfam of the docking and dimerization domain of protein kinase A II-alpha (R2D2, PF02197) (Fig. 1A). In order to test whether this domain could be important for the function of zC2orf62, we injected embryos with mRNA coding for the zC2orf62 C-terminal part (aa 276–356) and observed them at 24 hpf. As shown in figure S6D, about 75% of injected embryos displayed similar features than embryos injected with C2orf62 MO, including axis length reduction, decreased head size, and curved-down body shape.

L13A and F36A mutations were shown to abolish PRKAR2B dimerization and binding to AKAP75 [55]. Because these residues are conserved in C2orf62 (Fig. 1B), we mutated corresponding zC2orf62 residues (Leu-318 and Phe-341) into alanines and performed mRNA injection experiments. In contrast to injection of wild type C-terminus mRNA, injection of L318A/F341A C-terminus mRNA only had minor effect, since 80% of embryos injected with the mutant construct were normal (Fig. S6D). Taken together, these data suggest that zC2orf62 is involved in ciliogenesis through its R2D2-like domain.

## Discussion

In this report, we show that zC2orf62 expression is predominant in tissues rich in motile cilia (Kupffer’s vesicle, testis, lung, kidney) and in tissues rich in sensory/immotile cilia (olfactory pits, eye, ears, brain). The distribution of C2orf62 in mammalian tissues was similar, with highest expression levels found in testis. Expression of C2orf62 in human cell lines correlated with their ability to form cilia upon serum starvation. These observations suggest that C2orf62 could be associated with ciliogenesis.

Cilia/flagella are ancestral eukaryotic structures. Whereas proteins involved in their core assembly are conserved across species, regulatory proteins appeared in a stepwise manner during evolution [40] [1]. For example, CCP110, a centriolar protein that negatively controls the first steps of primary ciliogenesis [56], is conserved in metazoans but absent in *C. elegans* [57]. Likewise, C2orf62 is absent in *C. elegans*, suggesting a regulatory role in ciliogenesis rather than a direct function in cilia assembly. This is corroborated by the fact that, in ciliated human cells, C2orf62 is not localized on the cilium itself. Furthermore, in mammalian testis, C2orf62 is not present in mature, flagellate sperm cells, but in spermatocytes and in round and elongating spermatids where flagella formation is initiated [58]. These results suggest that C2orf62 would be important for processes occurring prior to or during cilia formation rather than for cilia maintenance or motility.

The early steps of primary ciliogenesis consist in the transfer of the basal body to the plasma membrane in a process that depends on the actin network [11]. Known regulators of actin polymerization have been recently shown to act on ciliogenesis [17] [47], and an inhibitory role of branched F-actin in ciliogenesis through modulation of ciliogenic vesicle trafficking is now recognized [48]. In human cells, C2orf62 was found to interact with TTC17, another uncharacterized protein. Down-regulation of either protein in hTERT-RPE1 or PANC-1 cells reduces the number of ciliated cells upon serum starvation while promoting actin polymerization. These effects were not seen in presence of cytochalasin D, an F-actin destabilizer known to induce ciliogenesis. These results provide evidence that C2orf62 acts together with TTC17 to favor primary ciliogenesis by negatively regulating actin polymerization.

Interestingly, in *Xenopus* embryos, c2orf62 was recently found to be strongly upregulated (9.3 fold) by multicilin (MCI), a protein required for multiciliate cell formation in diverse tissues [59]. Other genes found to be upregulated in this screen include foxj1, which is required for motile ciliogenesis and left-right patterning [60], its downstream genes tektins [61] and CCDC78 [61] [62], and several genes encoding centriole components. This observation suggests that C2orf62 may be involved in the regulation of motile ciliogenesis as well.

Zebrafish embryos depleted of either C2orf62 or TTC17 display a set of morphological features typical for imperfect motile ciliogenesis such as curly tail, heart positioning defects and cardiac edema [54] [53] [52] as well as a reduction in the number of primary ciliated cells in the olfactory organ. Thus, C2orf62 and TTC17 probably act on both motile and primary cilia formation.

In contrast to C2orf62, TTC17 is not exclusively distributed in human ciliated cells. TTC17 was found to be ubiquitously expressed in mammals, suggesting that it probably mediates additional cellular functions. Our preliminary data indicate that TTC17 expression is increased in human cells during mitosis. Although siRNA against TTC17 had no gross effect on cell proliferation rates, we cannot exclude that TTC17 may be involved in a cellular process that is linked to cell division. In addition, we showed that zC2orf62 was expressed in the maternal and early zygotic developmental periods of zebrafish, at a time when there is no ciliogenesis but extensive cell divisions [32]. In human cells, siRNA against C2orf62 slightly decreased cell proliferation rates, similarly to siRNA against MAPRE1. Therefore, we cannot exclude that some of the phenotypes induced by C2orf62 or TTC17 MOs in Zebrafish embryos could be due to cell proliferation defects. It will be particularly important to test the effects of C2orf62 or TTC17 MOs at 0–4 hpf in other F-actin-dependent processes such as gastrulation and epiboly [63] [64] [65] [66].

Injection of mRNA coding for the C-terminal part of zC2orf62 containing the R2D2-like domain induced the same morphological features at 24 hpf than injection of MOs against zC2orf62, suggesting an important function for the R2D2-like domain. R2D2 domains are found at the N-terminus of PKA regulatory subunits, and at the N-terminus of four proteins conserved in ciliated organisms and highly expressed in testes and ciliated cells: CABYR, ROPN1, ROPN1L/ASP and SPA17 [28] [29]. R2D2 domains homodimerize and interact with a short amphipathic helix motif located on AKAP proteins [67] [29]. R2D2 domains of PKA RII alpha and RII beta have been shown to target these proteins to the centrosome, microtubules and actin via interaction with AKAPs [68]. Peptides that disrupt interactions between R2D2 domains and AKAPs impair sperm motility [69], suggesting an important function for R2D2 domain-containing proteins in cilia or flagella function. This was recently confirmed for ROPN1 and ROPN1L [70] [71].We showed that mutations of Leu-318 and Phe-341, predicted to be required for dimerization and AKAP binding based on homology with other R2D2 domains [55] [67], prevented the developmental effects induced by the mRNA coding for the C-terminal part of zC2orf62. Therefore, a functional R2D2-like domain seems to be required for C2orf62 action on ciliogenesis in vivo, although we did not find any interaction between C2orf62 and an AKAP. The precise mechanism of action of this atypical C-terminal R2D2-like domain in ciliogenesis processes remains to be investigated. It could regulate C2orf62 oligomerization state and interaction with other proteins such as TTC17, or target C2orf62 to specific subcompartments enriched in cytoskeletal elements.

Taken together, our observations allow us to add C2orf62 and TTC17 to the growing list of genes directly or indirectly involved in the regulation of the formation of primary and motile cilia in Vertebrates. Due to the versatile role of cilia in human development and physiology, cilia defects can cause multiple severe diseases. Defects in motile cilia can lead to embryonic death, hydrocephalus, heart heterotaxy, or to primary ciliary dyskinesia (PCD), a complex disease with respiratory dysfunction and reproductive sterility, whereas defects in sensory cilia can result in altered development of limbs, polydactyly, retinal degeneration, nephropathies and cognitive impairement [72]. The molecular causes of motile and sensory ciliopathies are still incompletely defined, which complicates their diagnosis. For example, known mutations only account for 50% of all PCD cases. Based on our results, we propose to add C2orf62 and TTC17 to the list of candidate genes for ciliopathies of unknown etiology.

## Materials and Methods

### Zebrafish maintenance and cell culture

Zebrafish animal experimentation was approved by the Ethical Committee for Animal Experimentation of the Geneva University Medical School and the Canton of Geneva Animal Experimentation Veterinary authority. Wild-type (WT) AB and Casper [73] zebrafish were maintained in standard conditions (27°C, 500 µS, pH 7.5). Embryos obtained by natural intercrossing were staged according to morphology [32]. Ovaries and testes were excised from 3 month-old zebrafish euthanized using Tricaine.

Human cells were grown at 37°C in 5% CO2 in either DMEM with glutamax (HEK293T, HeLa, HepG2 and PANC-1) or DMEM/F12 (hTERT-RPE1) [74], and supplemented with 10% heat-inactivated fetal bovine serum (Gibco, 10270).

### Antibodies and staining reagents

Immunofluorescence studies on embryos were performed using rabbit anti-GFP (1∶500, Life Technologies), rabbit anti-pericentrin (1∶1000, Abcam, ab4448), mouse anti-acetylated tubulin (1∶250, Sigma, T7451), and goat anti-rabbit and anti-mouse antibodies (1/400, Life Technologies). Immunofluorescence studies on cells were performed using mouse anti-acetylated tubulin (1∶5000), anti-V5 (1∶500), anti TTC17 (1∶150) and goat anti-mouse and anti-rabbit (1/600) antibodies. Nuclei were stained with 4',6-Diamidino-2-Phenylindole (DAPI), and F-actin with Alexa-fluor 594 phalloidin (1/300, Life Technologies).

Immunohistochemistry experiments were performed using rabbit anti-C2orf62 and anti-TTC17 (1∶1000, HPA044818 and HPA038508, kind gifts of M. Uhlen), and non-immune serum (1∶1000) as negative control.

### Plasmids

C2orf62 cDNA was obtained from Life Technologies (IOH28795). PRKRA, CEP192 (aa 1501-1941) and TTC17 (aa 945-1041) cDNAs were generated by PCR on a mixture of human cDNA (Clontech). cDNAs were subcloned into pENTR/SD/D-TOPO or pENTR/TEV/D-TOPO (Life Technologies) and transferred into EGFP, mCherry, GST or V5 pDEST plasmids using Gateway BP Clonase II. For yeast two-hybrid, the C2orf62 cDNA was cloned into pDBa.

zC2orf62 cDNA obtained by PCR on a mixture of embryo cDNAs was subcloned into pCS2^+^, pCS2^+^ EGFP and pCRII TOPO plasmids (Life Technologies). The 3′ and 5′-flanking zC2orf62 DNA regions were amplified by PCR. The reporter plasmid was constructed by inserting EGFP between the 2.8 kb upstream and 0.4 kb downstream sequences of zC2orf62 in pT2KXIG▵in (Fig. 2C).

zC2orf62 C-ter (aa 276–356) cDNA was obtained by PCR from zC2orf62 pCS2+. L318A/F341A zC2orf62 C-ter (aa 276–356) cDNA was obtained by PCR overlap extension using primers harboring the mutations. Both cDNAs were cloned into pCS2+ vector.

### Genome-wide expression profiling

The Affymetrix Rat Exon 1.0 ST GeneChip dataset includes six enriched populations of testicular cell types (Leydig, peritubular myoid and Sertoli cells, spermatogonia, pachytene spermatocytes and round spermatids) in triplicates, complemented with twelve tissues: ovary, bone marrow, brain, embryo, heart, kidney, liver, lung, muscle, spleen, thymus (3 samples each), and testis (7 samples). GeneChip data were normalized using the Robust Multi-Array Average method [75]. The RNA-seq dataset (Illumina's protocol) includes Sertoli cells, spermatogonia, pachytene spermatocytes and round spermatids (in duplicates). The Tuxedo Suite [76] was used to map RNA-seq-derived reads on the genome, and to assemble and quantify transcripts.

### RT-PCR and RT-qPCR

RNA extracted with RNeasy Qiagen columns was treated with DNase (Ambion) and checked with an Agilent Bioanalyser. Reverse transcription was carried out from 2 µg RNA using random hexamers (Promega) and SuperScript III Reverse Transcriptase (Life Technologies). The thermal profile used for PCR on human cells and tissue cDNAs (Clontech) was: 95°C for 20 s, 58°C for 40 s and 72°C for 34 s, 37 cycles.

q-PCR on zebrafish cDNAs was performed in triplicates in 10 µl samples containing 1μl SYBR green reagent, 200 nM oligonucleotides and 1/20 of total cDNA (50°C for 2 min, 95°C for 10 min, 40 cycles of 95°C for 15 s and 60°C for 1 min) using either Amplicon 1 primers zC2orf62_335F (TGGAGCAGTGTTTGTTTGCAG) and zC2orf62_385R (TGCCTGAATCAGACACGGTC) or Amplicon 2 primers zC2orf62_186F (AGCCTGTTGAAGGATTAAGCTGTTA) and zC2orf62_263R (TGAGTTAATTCACTTTCCTCCATGTC).

Relative levels of RNAs were calculated on the basis of ▵CT (Cycle Threshold variation) and normalized to the geometric mean of *beta-actin*, *ef1-alpha* [77] and *odc1* RNA levels.

### Microinjection

C2orf62_1 MO (5′-GTGCTGCAAATGACAGCATAAGTGA-3′), C2orf62_2 MO (5′-CTTTCCTCCATGTCTTAAAAACTCC-3′), TTC17_1 MO (5′- GACACACTCGCTCACCTGCTGCTGT-3′), TTC17_2 MO (5′- GAACATGAGGGTTAAAATCACCTCT-3′) and Ctrl MO (5′-CCTCTTACCTCAGTTACAATTTATA-3′) (Gene Tools) were dissolved in nuclease-free water and their concentrations determined with NanoDrop.

zC2orf62 mRNA was in vitro transcribed from the zC2orf62 pCS2^+^ plasmid using the mMessage mMachine System (Ambion) and purified by phenol/chloroform extraction.

MO and mRNA were injected at 1–2 cell stages using 0.1% phenol red in 0.5–2 nl Danieau buffer.

To generate EGFP-zC2orf62 reporter fish, AB and Casper embryos were microinjected with 1 nl of a solution composed of 35 ng/µl Tol2 transposase mRNA, 25 ng/µl EGFP reporter plasmid, and 0.1% phenol red as described [78], resulting in generation number 0 (G0). G1 and G2 embryos were established by successively mating adults with WT fish. EGFP-zC2orf62 carriers were identified by fluorescence stereomicroscopy, and imaged using either a Leica DFC340FX digital camera with Leica software LAF 2.1 or a Zeiss LSM 510 META confocal microscope with LSM Viewer software. ImageJ (rsb.info.nih.gov/ij/) was used for image processing.

### Whole-mount in situ hybridization

RNA probes for zC2orf62, *shha*, *ntla* and *myh6* were in vitro transcribed with SP6 and T7 RNA polymerases (Promega) from linearized pCRII TOPO plasmids, and labeled using DIG RNA labeling kit (Roche). Dechorionated embryos were fixed overnight with 4% paraformaldehyde in PBS at 4°C and stored in methanol at –20°C. Embryos were rehydrated in PBS/methanol, washed in 0.1% Tween 20, treated with H2O2 during 30 min, and permeabilized using Proteinase K (10 µg/ml, Roche). Embryos were hybridized overnight at 50°C, washed in increasing concentrations of methanol, and clarified in glycerol as described [79]. Imaging was performed using a MZ16FA stereomicroscope and a DFC420 camera (Leica).

### Immunohistochemistry

Immunohistochemical experiments were performed on testes from 90 dpp male Sprague-Dawley rats and on human testes, fixed in Bouin’s fixative and embedded in paraffin, as described [80]. Human testes were obtained from patients undergoing therapeutic orchidectomy for metastatic prostate carcinoma. The protocol was approved by the Ethical Committee of Rennes, France (Authorization n°DC-2010-1155 - June 15 2011) and written informed consent was obtained from all donors. Thin sections (5 µm) were deparaffined, rehydrated, and incubated for 1 hr at 80°C in citrate buffer (10 mM pH 6.0) with 0.05% Tween 20 for antigen retrieval. After saturation for 30 min with 1% BSA in PBS, the sections were incubated overnight at 48C with the rabbit polyclonal anti-C2orf62 or anti-TTC17 antibodies used both at a final dilution of 1∶1000, in PBS containing 0.1% Tween-20 (v/v) and 1% BSA (PBST-BSA). After several washes in PBS, sections were incubated for 45 min with a secondary biotinylated mouse anti-rabbit antibody (Dako, Trappes, France) at a final dilution of 1∶500 in PBS-BSA. Samples were subsequently washed in PBS and incubated for an additional 30 min with a streptavidin-peroxidase complex (Dako) at a dilution of 1∶500 in PBS. Immunoreaction was revealed with a diaminobenzidine solution (Sigma). Finally, sections were counterstained with Masson hemalun, dehydrated, and mounted in Eukitt (Labnord, Villeneuve d’Asq, France).

### Cell transfection and imaging

Cells grown on coverslips were transfected with plasmids using Fugene (Roche), and 6 hr later with siRNA (Ambion) against C2orf62 (5′-AGACCAUCCAGGUAGACCAtt-3′; s51680), TTC17 (5′-CAGUGAUGAUUAUUCUACAtt-3′; s31447), MAPRE1 (5′-CCUGUGGACAAAUUAGUAAtt-3′; s22674) or with control siRNA (AM-4611) using lipofectamine RNAiMAX Reagent (Life Technologies). Serum starvation was performed 24 hrs later during 72 hr (PANC-1) or 48 hr (hTERT-RPE1). Cells could be treated during the last 8 hrs with 0.5 uM Cytochalasin D (Sigma C8273) or DMSO**.**

Cells were fixed for 20 min in 4% paraformaldehyde, treated with NH_4_Cl during 20 min, permeabilized with 0.1% Triton X-100 in PBS (PBS-T) for 20 min, and incubated in PBS-T with 3% BSA during 1 hr. The coverslips were incubated overnight at 4°C with the primary antibodies, washed in PBS-T, incubated with secondary antibodies, and mounted on slides.

Quantification of cilia was performed on randomly selected cells, based on acetylated tubulin labeling. ImageJ was used for image processing. Cilia lengths were measured following acquisition of *z*-stacks. 3D depictions of cilia were reconstructed using Imaris (Bitplane scientific software).

To estimate hTERT-RPE1 cell proliferation rates, equivalent numbers of cells were plated in 12 well plates, transfected with siRNA 24 hours later, and counted using a hemocytometer 48 or 72 hours after the transfection.

For time-lapse imaging, cells were cultured in 96 well black plates with clear bottom (Costar 3603) and transfected with plasmids using Fugene. Imaging was performed every 2 min during 16 hrs on ImageXpress Micro from Molecular Devices equipped with transmitted light at 37°C and 5% CO_2_.

For FRET experiments, cells were cultured in individual culture dishes (Fluorodish FD35–100) and transfected with plasmids using Xtreme gene (Roche). Imaging was performed in a controlled atmosphere chamber at 60X magnification under oil immersion, using a Nikon A1R confocal microscope running with NIS element AR imaging software v.4.11.01. Using ImageJ, images were successively corrected for background, realigned, converted in 32 bits, and smoothed as described [81]. Then, a threshold and a ratio (mCherry/GFP) were applied. FRET signals were corrected for bleed-through by measuring the donor spectral bleed-trough into the acceptor channel and the direct acceptor excitation, following three spectral configurations as described (Padilla-Parra and Tramier, 2012), and presented as normalized FRET (FRETn). Pseudocolors images were generated on the basis of 16 colors, with the lowest FRET intensity in black and the highest FRET intensities in white and red.

### Yeast two-hybrid

The C2orf62 pDBa (Leu) plasmid was transformed in MaV203 yeast strain (MATα; leu2-3,112; trp1-901; his3▵200; ade2-101; gal4▵, gal80▵ SPAL10UASGAL1::URA3, GAL1::lacZ, GAL1::His3@LYS2, can1R,cyh2R) as described [82]. This bait did not show self-activation and was further used for screening. MaV203 cells were transformed with human fetal brain cDNA library (cloned into pEXP502-AD (Trp), Proquest libraries™, Life Technologies), plated onto Synthetic Complete (SC) medium minus Leucine (-L), minus Tryptophan (-W), minus Histidine (-H) + 25 mM 3-amino-1,2,4-triazole (3-AT), and incubated at 30°C for 4–5 days. Positive clones were patched onto SC-WHL + 3-AT in 96-well plates, incubated for 3 days at 30°C and transferred in liquid SC-WL for 3 days at 30°C with agitation to normalize the yeast cell concentration used for the phenotypic assay. Cells were then diluted 1/20 in water, spotted onto selective medium (-WHL+25 mM 3-AT or -WUL) and incubated at 30°C for 4 to 5 days. To perform the β-galactosidase assay, undiluted yeast cells were spotted onto YPD (yeast extract peptone dextrose) medium plates with nitrocellulose filters, and β-galactosidase activity was evaluated one day after. Only the interactors that were positive for the three phenotypes tested (growth on -WHL+25 mM 3-AT, or -WUL or β-galactosidase) were further analyzed.

### GST pull down

48 hrs after transfection with C2orf62 pDEST-V5, HEK293T cells were lysed in extraction medium (0.1 M PIPES pH 6.8; 5 mM MgCl_2_; 150 mM NaCl, 1% Nonidet P40) containing protease inhibitors (Roche).

*E. coli* BL21 were transformed with the PRKRA-GST, TTC17-GST and CEP192-GST plasmids by heat shock. GST fusion proteins were induced by 1 mM IPTG and extracted by sonication (4 times 15 s) in lysis buffer (10 mM Tris-HCl (pH 8.0); 5 mM MgCl_2_; 0,15 M NaCl; 1% Triton; 5 mM DTT). After centrifugation at 10,000 *g* for 10 min, the supernatants were incubated with swelled glutathione–agarose beads (Sigma, G4510) during 60 min before extensive washings. V5-C2orf62 cell lysates previously cleared on beads (1 mg protein) were incubated on GST-fusion proteins beads for 2 hrs at 4°C.

Following SDS–PAGE, bound proteins were stained with Coomassie or analysed by Western blot using mouse anti-V5 antibody (AbD Serotec; 1∶1000) and alkaline phosphatase conjugated goat anti-mouse antibody (Jackson ImmunoResearch; 1∶5000).

### Sequence analysis

BLASTP and TBLASTN analysis were performed on UniProtKB (UniProt consortium, 2012) release 2012_07 and the NCBI Reference Sequences (RefSeq) [83]. Multiple sequence alignments were performed using MUSCLE 3.6 with the default parameters [84]. C2orf62 and zC2orf62 sequences were aligned on genomes using BLAT [85] on the UCSC genome browser (genome.ucsc.edu). Domain analysis was performed using Pfam [26].

### Statistics

Statistical analyses were performed with Microsoft Excel. Error bars are means +/– s.d. of values from at least 3 independent experiments. Statistical significance (P-value) was calculated using a two-tailed paired t-test (for rescue of MO effects), or a two-sample with unequal variance t-test (for experiments on cells), and is indicated by * *P*<0.05, ** *P*< 0.01, ***P<0.001 or n.s. (not significant).

## Supporting Information

Figure S1 **(related to** **Fig. 2** **).** **zC2orf62 expression pattern during development.** Reverse Transcription-quantitative Polymerase Chain Reaction (*RT*-*qPCR*) performed with Amplicon 2 of zC2orf62 shows the same expression pattern than in Fig. 2. zC2orf62 mRNA is expressed essentially in testis (100 times more than in ovary), but also during almost all development. It is down-regulated at shield stage (6 hpf) and reexpressed at tail bud (12 hpf) when Kupffer's vesicle forms.
(TIF)

Figure S2 **(related to** **Fig. 2** **). zC2orf62 is expressed in ciliated cells during embryonic development.** Transgenic EGFP-zC2orf62 reporter zebrafish were observed using a confocal microscope. (**A**) At 48 hpf, immunostaining of α-acetylated tubulin (red) and EGFP (green) show a co-localization (yellow) in olfactory placode (OP), neuromast cells (N), ear (E) and pronephric ducts (P.D), and in cilia of olfactory sensory neurons (OSN). (**B**) At 96 hpf, EGFP is expressed specifically in ciliated neuromast cells and in hair cell-containing structures of the ear (cristae and macula). Scale bars, 25 µm.
(TIF)

Figure S3 **(related to** **Figs. 3** **and** **4** **). Analysis of TTC17 expression in rat and human tissues and cell lines.** (**A**) Histograms displaying Ttc17 transcript levels in rat somatic testicular cells [Leydig (LE), peritubular myoid (PM) and Sertoli (SE)], male germ cells [spermatogonia (SG), spermatocytes (SC) and spermatids (ST)] and twelve normal tissues based on the Affymetrix Rat Exon 1.0 ST (left) and Illumina RNA-seq (right) datasets. (**B**) Transverse sections of adult rat testis (left) and human testis (right) showing immunoreactivity in cells with TTC17 antibody. In rat, TTC17 is detected in the germ cell lineage from preleptotene spermatocytes to elongating spermatids, here at stage II-III of spermatogenesis (left panel). Please note the strong staining in both the Leydig cells and capillary endothelium. TTC17 is detected in the cytoplasm of pachytene spermatocytes (SPC), early (rSPT) and elongating spermatids (eSPT) at stage II-III of spermatogenesis (right panel). In human, TTC17 is detected in the cytoplasm of spermatocytes and spermatids. Inserts**:** negative controls using preimmune serum. Scale bars, 50 µm. (**C**) Subcellular localization of endogenous TTC17 was studied by immunofluorescence on hTERT-RPE1 cells transfected either with a control siRNA (si Ctrl.) or with siRNA against TTC17 (si TTC17). Despite a strong background staining in the nucleus, a specific staining (arrows) that disappeared with si TTC17 treatment could be seen in non-dividing cells. This staining was considerably enhanced during mitosis, from metaphase to telophase. Scale bars, 10 µm.
(TIF)

Figure S4 **(related to** **Fig. 5** **). Effects of C2orf62 siRNA knockdown on cell proliferation and actin polymerization. (A)** hTERT-RPE1 cells were counted using a hematocytometer 48 h and 72 h after transfection with the indicated siRNA. The bar graphs show the ratios of proliferation rates of cells transfected with siRNA against MAPRE1, C2orf62 or TTC17 as compared to cells transfected with control siRNA, measured in 4 independent experiments. None of the tested siRNA affected cell proliferation when it was assayed 48 h after transfection, Both si C2orf62 and si MAPRE1 slightly inhibit cell proliferation when it was assayed 72 h after transfection (C2orf62 siRNA/ Ctrl siRNA *P = 0.034; MAPRE1 siRNA/Ctrl siRNA *P = 0.031). (**B**) PANC-1 cells were transfected with siRNA against C2orf62 or with a control siRNA. Cells were either serum-starved for 72 hours (0.5% FBS) or left in 10% FBS medium, and F-actin was stained using Alexa fluor 594 phalloidin. Scale bars, 50 µm. C2orf62 knockdown results in enhanced actin polymerization in both serum conditions.
(TIF)

Figure S5 **(related to** **Fig. 6** **).** **Evaluation of the specificity of C2orf62 morpholinos**. Zebrafish embryos were injected with different quantities of C2orf62_1 or C2orf62_2 MO and observed under a stereomicroscope. (**A-C**) Phenotypic traits at 24 hpf were classified into four categories, each being associated with one grey code, as reported in Fig. 6A: normal (white, indistinguishable from controls), severe (clear grey, defect of brain structure formation and short body axis), very severe (dark grey, indistinguishable left/right or front/back polarity), and lethal (black). The bar graphs show the percentage of embryos in each category. (**A**) Although the injection of 1.25 ng C2orf62_1 or C2orf62_2 MO has no effect on embryo morphology, the co-injection of 1.25 ng C2orf62_1 MO and 1.25 ng C2orf62_2 MO results in 47% of severe-to-lethal phenotypes, showing a strong synergy between both MOs. (**B**) Embryos were injected with 5 or 10 ng C2orf62_2 MO together with 0 (buffer alone), 15 or 30 ng P53 MO. Coinjection of P53 MO does not significantly change the phenotypes induced by injection of C2orf62_2 MO alone. (**C**) Embryos were injected with 10 ng C2orf62_2 MO and/or 2 ng full-length zC2orf62 mRNA. The percentage of normal (white) C2orf62_2 MO-injected embryos is significantly higher in the presence of mRNA (1.96% for C2orf62_2 MO alone and 12.53% for C2orf62_2 MO + RNA, P = 0.0044, Student's *t* test). (**D**) Embryos were co-injected with 680 pg C2orf62-EGFP *mRNA* and 5 ng Ctrl. MO, C2orf62_1 MO or C2orf62_2 MO and observed 6 h later under a fluorescence stereomicroscope. EGFP signal was abrogated in all C2orf62_1 MO-injected embryos (star marked), and unexpectedly in 33% of C2orf62_2 MO-injected embryos. (**E**)**.** RT-PCR analysis of total RNA extracted from 24 hpf embryos after injection of 5 ng of Ctrl. MO or C2orf62_2 MO shows that C2orf62_2 MO alters zC2orf62 splicing.
(TIF)

Figure S6 **(related to** **Fig. 6** **). Knockdown of zC2orf62 results in notochord undulation and ciliogenesis defects in the olfactory organ.** (**A**) In situ hybridization of *ntla and shha* at 24 hpf shows that more than 90% of C2orf62_1 MO-injected embryos have an undulated notochord, in contrast to controls (lateral views, scale bars, 200 µm). (**B**) Embryos were either not injected (n.i.) or injected with 5 or 10 ng C2orf62_1 MO and observed at 48 hpf. C2orf62_1 MO-injected embryos display a curved body, heart edema (arrow), and a small head with incorrectly defined brain structures, small eyes and small ears (dashed circle). Scale bars, 200 µm. (**C**) Embryos were injected either with 2.5 ng Ctrl. MO or C2orf62_1 MO, and ciliated cells from the olfactory organ (OO) were visualized using anti α-acetylated tubulin at 48 hpf. The olfactory organs of C2orf62_1 MO-injected embryos are smaller and display less ciliated cells than controls. Scale bars, 50 µm. (**D**) Embryos were injected with 850 pg mRNA coding for the C-terminal part (aa 276-356) of zC2orf62,either wild type (WT C-ter) or alanine-mutated on Leu-318 and Phe-341 (L318A/F341A C-ter), and observed 24 h later. Phenotypic traits were classified into four categories, each being associated with one grey code, as reported in Fig. 6A: normal (white, indistinguishable from controls), severe (clear grey, defect of brain structure formation and short body axis), very severe (dark grey, indistinguishable left/right or front/back polarity), and lethal (black). The bar graph shows the percentage of embryos in each category. The percentage of normal (white) embryos is significantly higher for L318A/F341A C-ter mRNA-injected embryos than for WT C-ter mRNA-injected embryos (25.7% for WT C-ter mRNA and 80.6% for L318A/F341A C-ter mRNA P = 0.0078, Student's *t* test).
(TIF)

Table S1 **(related to** **Fig. 4** **). Yeast two-hybrid results**. 10 positive clones obtained in the yeast two-hybrid screen were identified by sequencing. Clones labeled as “Not relevant” are typical yeast two-hybrid artefacts.
(DOCX)

Movie S1 **(related to** **Fig. 4** **). EGFP-C2orf62 localization in hTERT-RPE1 cells.** The movie shows the dynamic localization of EGFP-C2orf62 (in green) in hTERT-RPE1 cells 2.25 hours after transfection. The EGFP-C2orf62 fusion protein is localized in the cytoplasm, nucleus and in plasma membrane protrusions.
(DOCX)
