# Supplementary figures and images for: C2orf62 and TTC17 Are Involved in Actin Organization and Ciliogenesis in Zebrafish and Human
Source: PLoS One. 2014 Jan 27;9(1):e86476. doi: 10.1371/journal.pone.0086476 (PMC3903541; doi:10.1371/journal.pone.0086476)

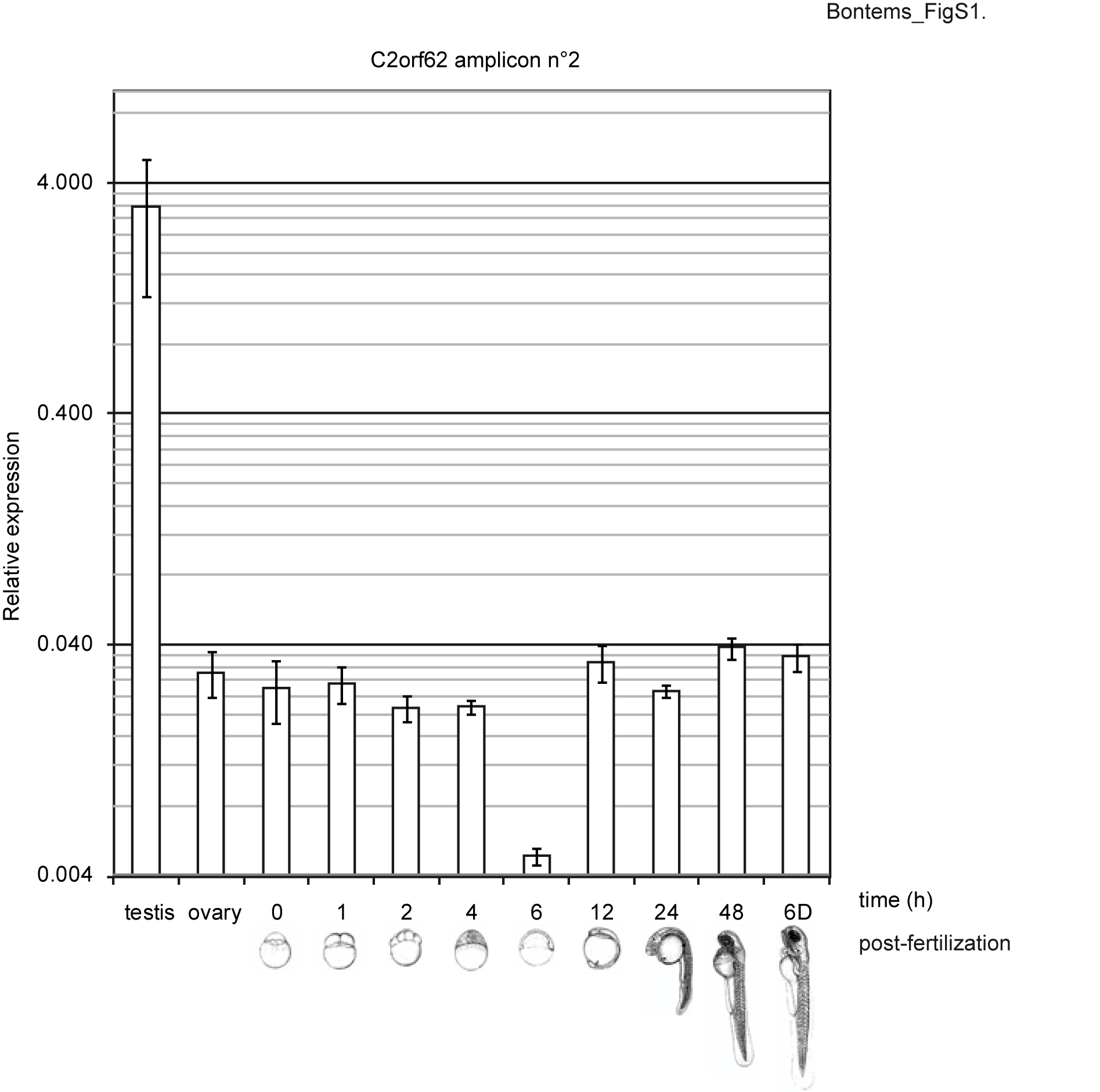

Supplement: Figure S1 — (related to Fig. 2 ). zC2orf62 expression pattern during development. Reverse Transcription-quantitative Polymerase Chain Reaction (RT-qPCR) performed with Amplicon 2 of zC2orf62 shows the same expression pattern than in Fig. 2. zC2orf62 mRNA is expressed essentially in testis (100 times more than in ovary), but also during almost all development. It is down-regulated at shield stage (6 hpf) and reexpressed at tail bud (12 hpf) when Kupffer's vesicle forms. (TIF) [file pone.0086476.s001.tif]

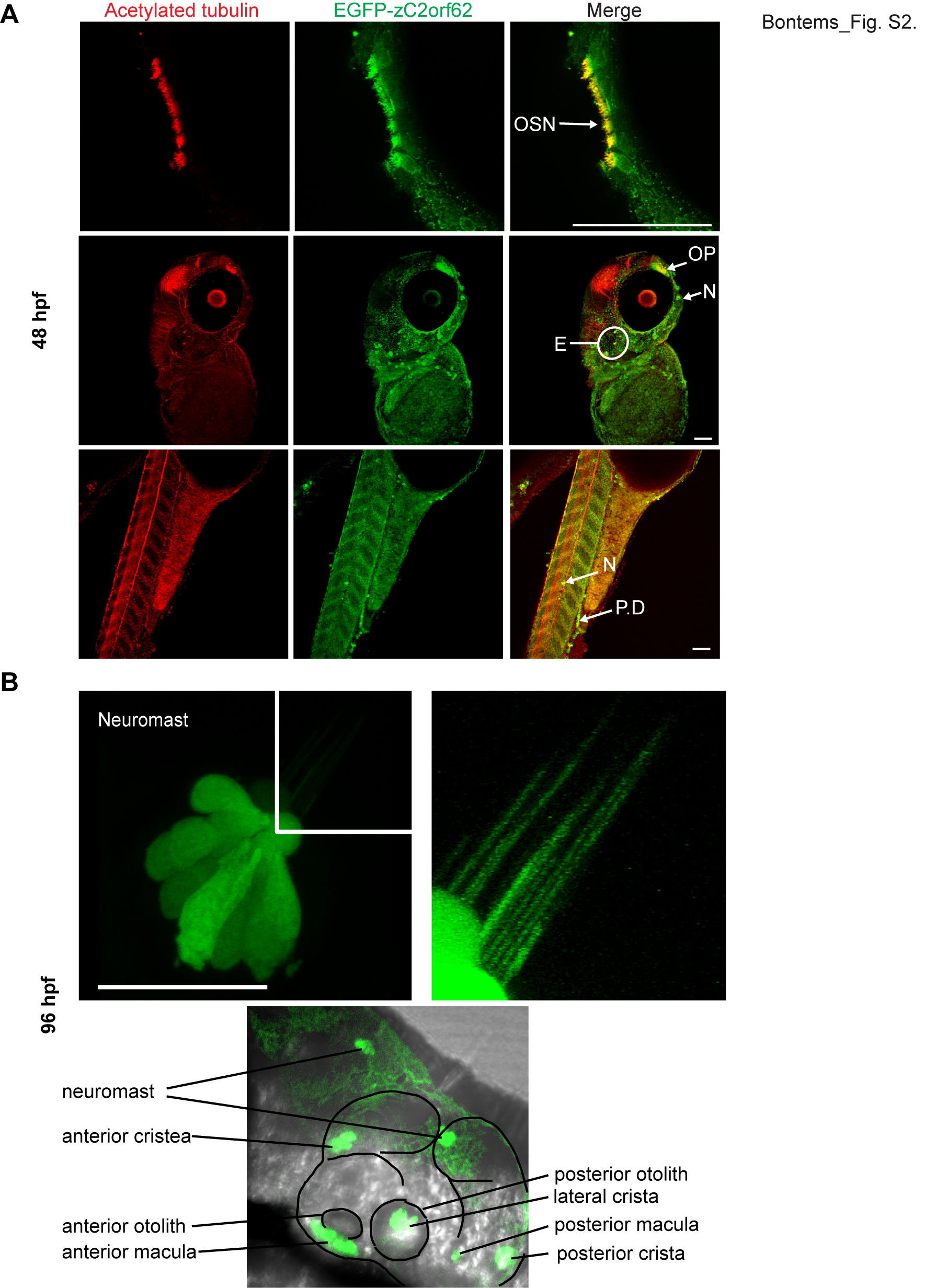

Supplement: Figure S2 — (related to Fig. 2 ). zC2orf62 is expressed in ciliated cells during embryonic development. Transgenic EGFP-zC2orf62 reporter zebrafish were observed using a confocal microscope. (A) At 48 hpf, immunostaining of α-acetylated tubulin (red) and EGFP (green) show a co-localization (yellow) in olfactory placode (OP), neuromast cells (N), ear (E) and pronephric ducts (P.D), and in cilia of olfactory sensory neurons (OSN). (B) At 96 hpf, EGFP is expressed specifically in ciliated neuromast cells and in hair cell-containing structures of the ear (cristae and macula). Scale bars, 25 µm. (TIF) [file pone.0086476.s002.tif]

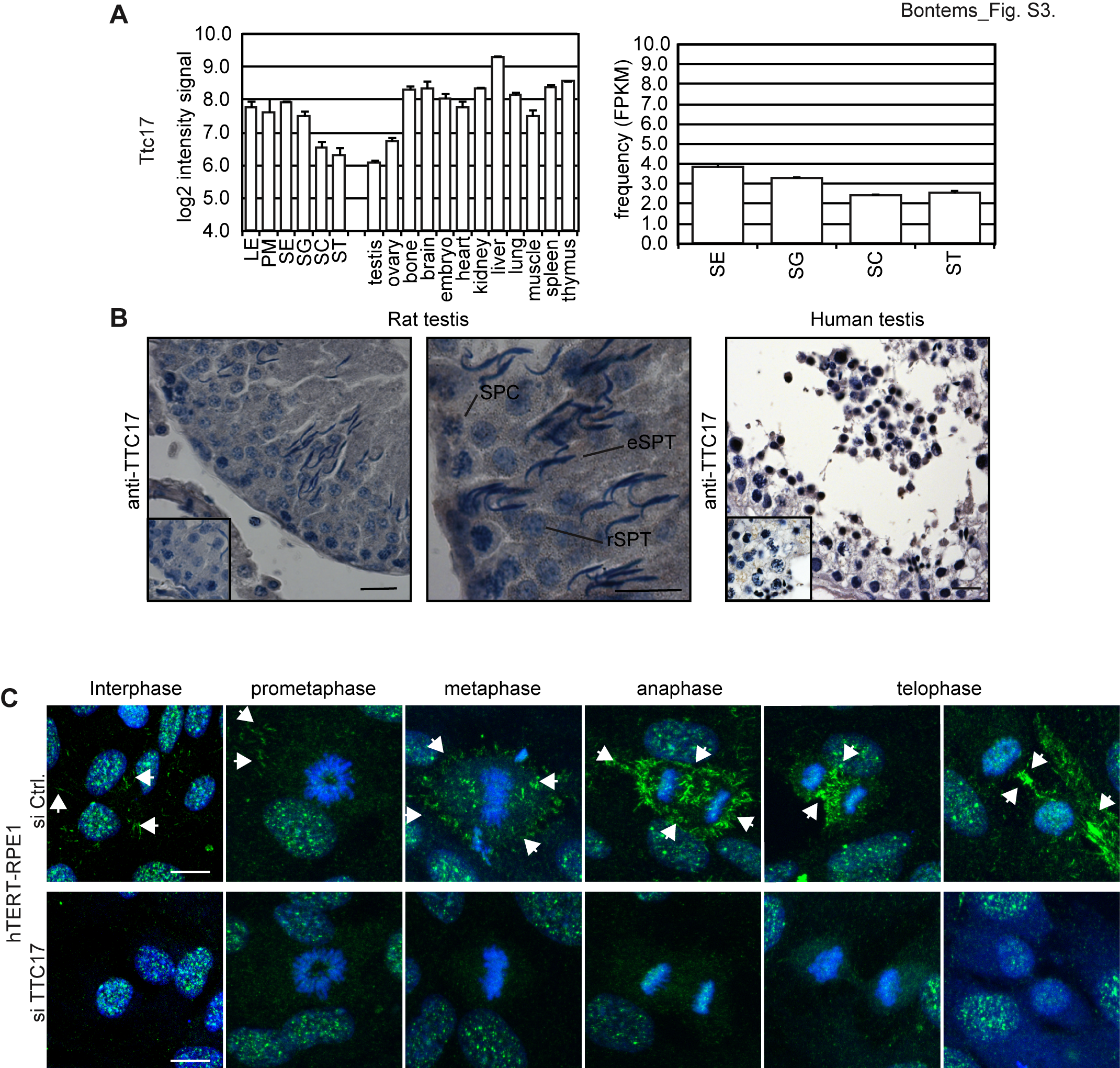

Supplement: Figure S3 — (related to Figs. 3 and 4 ). Analysis of TTC17 expression in rat and human tissues and cell lines. (A) Histograms displaying Ttc17 transcript levels in rat somatic testicular cells [Leydig (LE), peritubular myoid (PM) and Sertoli (SE)], male germ cells [spermatogonia (SG), spermatocytes (SC) and spermatids (ST)] and twelve normal tissues based on the Affymetrix Rat Exon 1.0 ST (left) and Illumina RNA-seq (right) datasets. (B) Transverse sections of adult rat testis (left) and human testis (right) showing immunoreactivity in cells with TTC17 antibody. In rat, TTC17 is detected in the germ cell lineage from preleptotene spermatocytes to elongating spermatids, here at stage II-III of spermatogenesis (left panel). Please note the strong staining in both the Leydig cells and capillary endothelium. TTC17 is detected in the cytoplasm of pachytene spermatocytes (SPC), early (rSPT) and elongating spermatids (eSPT) at stage II-III of spermatogenesis (right panel). In human, TTC17 is detected in the cytoplasm of spermatocytes and spermatids. Inserts: negative controls using preimmune serum. Scale bars, 50 µm. (C) Subcellular localization of endogenous TTC17 was studied by immunofluorescence on hTERT-RPE1 cells transfected either with a control siRNA (si Ctrl.) or with siRNA against TTC17 (si TTC17). Despite a strong background staining in the nucleus, a specific staining (arrows) that disappeared with si TTC17 treatment could be seen in non-dividing cells. This staining was considerably enhanced during mitosis, from metaphase to telophase. Scale bars, 10 µm. (TIF) [file pone.0086476.s003.tif]

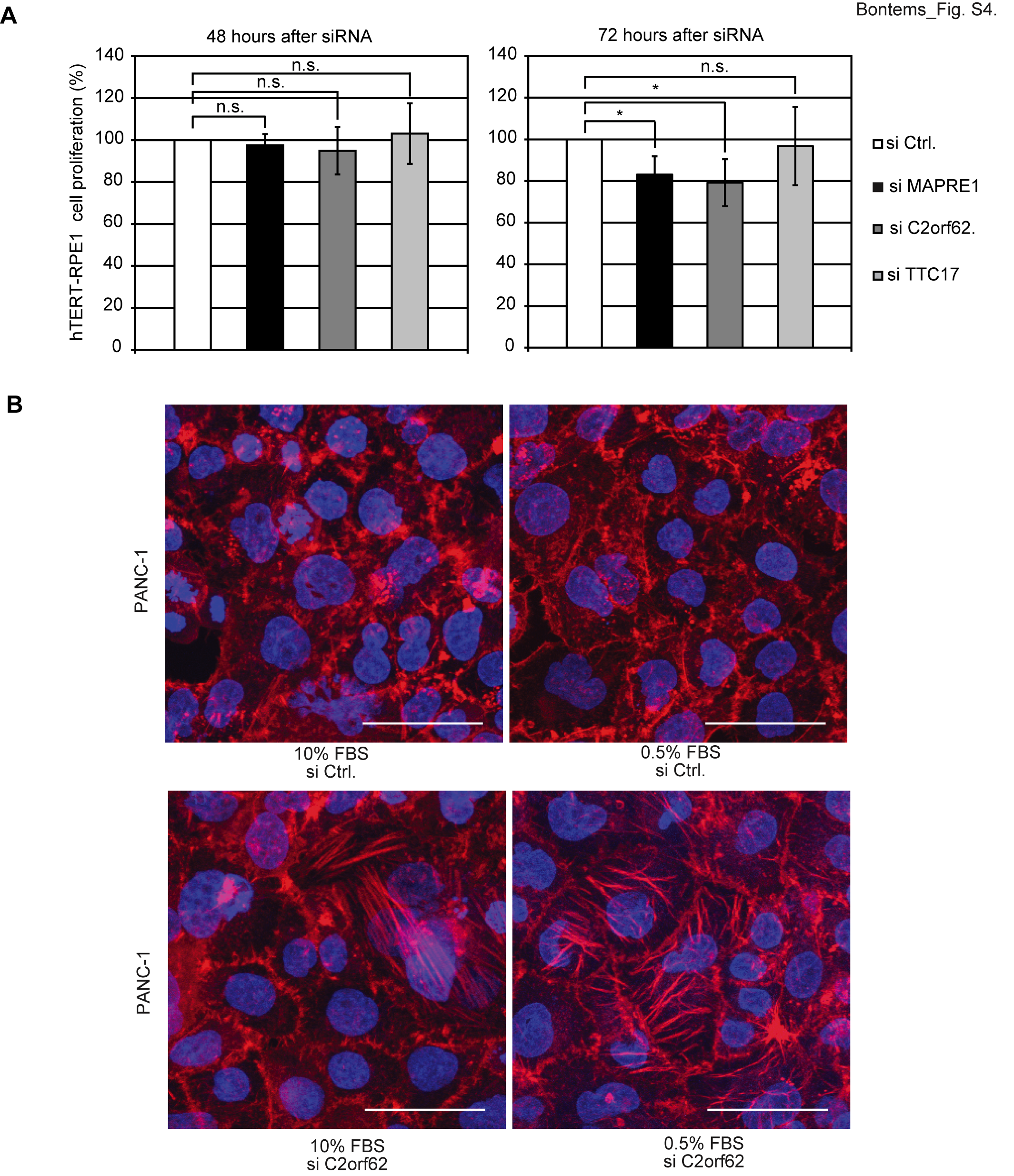

Supplement: Figure S4 — (related to Fig. 5 ). Effects of C2orf62 siRNA knockdown on cell proliferation and actin polymerization. (A) hTERT-RPE1 cells were counted using a hematocytometer 48 h and 72 h after transfection with the indicated siRNA. The bar graphs show the ratios of proliferation rates of cells transfected with siRNA against MAPRE1, C2orf62 or TTC17 as compared to cells transfected with control siRNA, measured in 4 independent experiments. None of the tested siRNA affected cell proliferation when it was assayed 48 h after transfection, Both si C2orf62 and si MAPRE1 slightly inhibit cell proliferation when it was assayed 72 h after transfection (C2orf62 siRNA/ Ctrl siRNA *P = 0.034; MAPRE1 siRNA/Ctrl siRNA *P = 0.031). (B) PANC-1 cells were transfected with siRNA against C2orf62 or with a control siRNA. Cells were either serum-starved for 72 hours (0.5% FBS) or left in 10% FBS medium, and F-actin was stained using Alexa fluor 594 phalloidin. Scale bars, 50 µm. C2orf62 knockdown results in enhanced actin polymerization in both serum conditions. (TIF) [file pone.0086476.s004.tif]

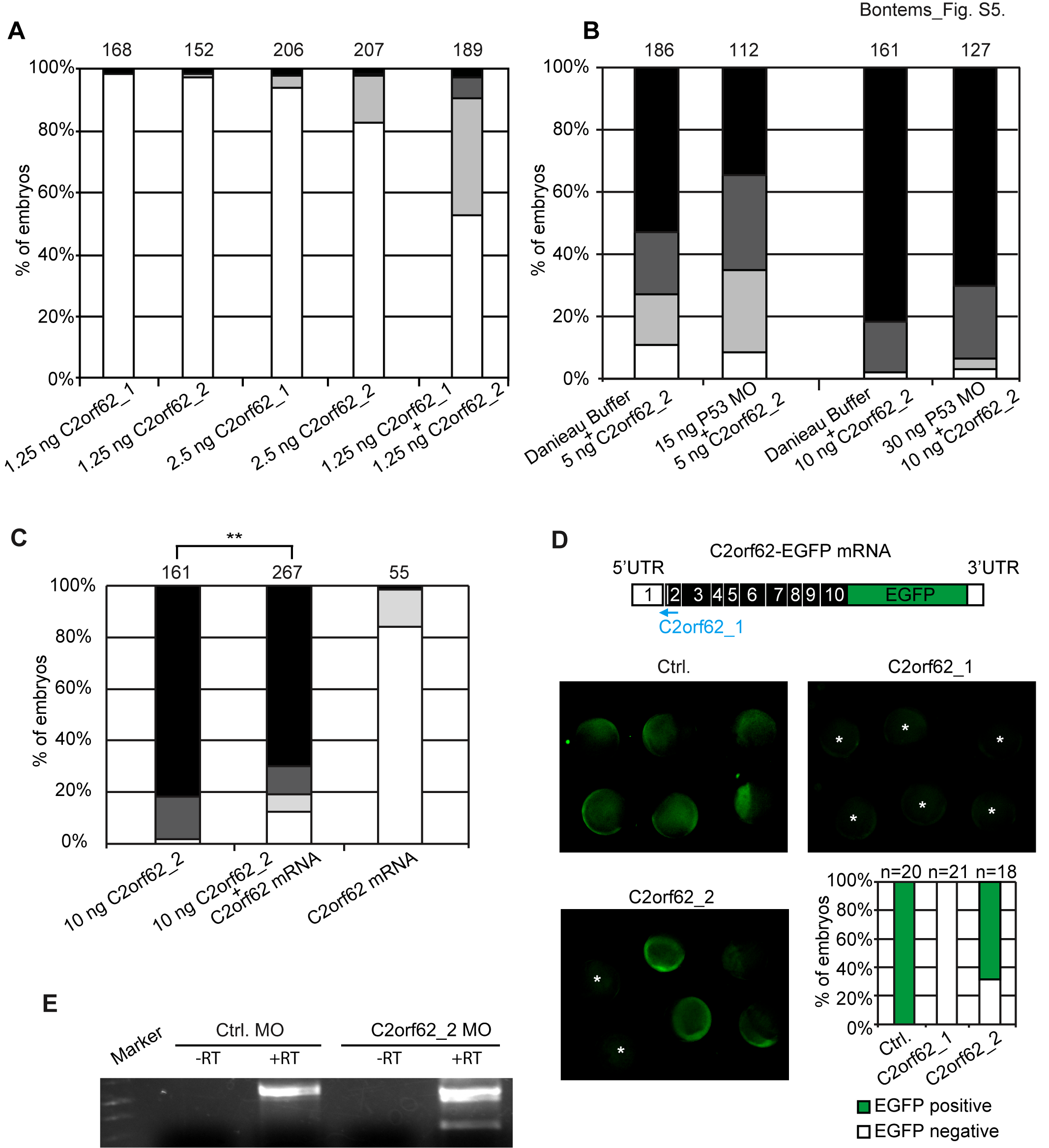

Supplement: Figure S5 — (related to Fig. 6 ). Evaluation of the specificity of C2orf62 morpholinos. Zebrafish embryos were injected with different quantities of C2orf62_1 or C2orf62_2 MO and observed under a stereomicroscope. (A-C) Phenotypic traits at 24 hpf were classified into four categories, each being associated with one grey code, as reported in Fig. 6A: normal (white, indistinguishable from controls), severe (clear grey, defect of brain structure formation and short body axis), very severe (dark grey, indistinguishable left/right or front/back polarity), and lethal (black). The bar graphs show the percentage of embryos in each category. (A) Although the injection of 1.25 ng C2orf62_1 or C2orf62_2 MO has no effect on embryo morphology, the co-injection of 1.25 ng C2orf62_1 MO and 1.25 ng C2orf62_2 MO results in 47% of severe-to-lethal phenotypes, showing a strong synergy between both MOs. (B) Embryos were injected with 5 or 10 ng C2orf62_2 MO together with 0 (buffer alone), 15 or 30 ng P53 MO. Coinjection of P53 MO does not significantly change the phenotypes induced by injection of C2orf62_2 MO alone. (C) Embryos were injected with 10 ng C2orf62_2 MO and/or 2 ng full-length zC2orf62 mRNA. The percentage of normal (white) C2orf62_2 MO-injected embryos is significantly higher in the presence of mRNA (1.96% for C2orf62_2 MO alone and 12.53% for C2orf62_2 MO + RNA, P = 0.0044, Student's t test). (D) Embryos were co-injected with 680 pg C2orf62-EGFP mRNA and 5 ng Ctrl. MO, C2orf62_1 MO or C2orf62_2 MO and observed 6 h later under a fluorescence stereomicroscope. EGFP signal was abrogated in all C2orf62_1 MO-injected embryos (star marked), and unexpectedly in 33% of C2orf62_2 MO-injected embryos. (E). RT-PCR analysis of total RNA extracted from 24 hpf embryos after injection of 5 ng of Ctrl. MO or C2orf62_2 MO shows that C2orf62_2 MO alters zC2orf62 splicing. (TIF) [file pone.0086476.s005.tif]

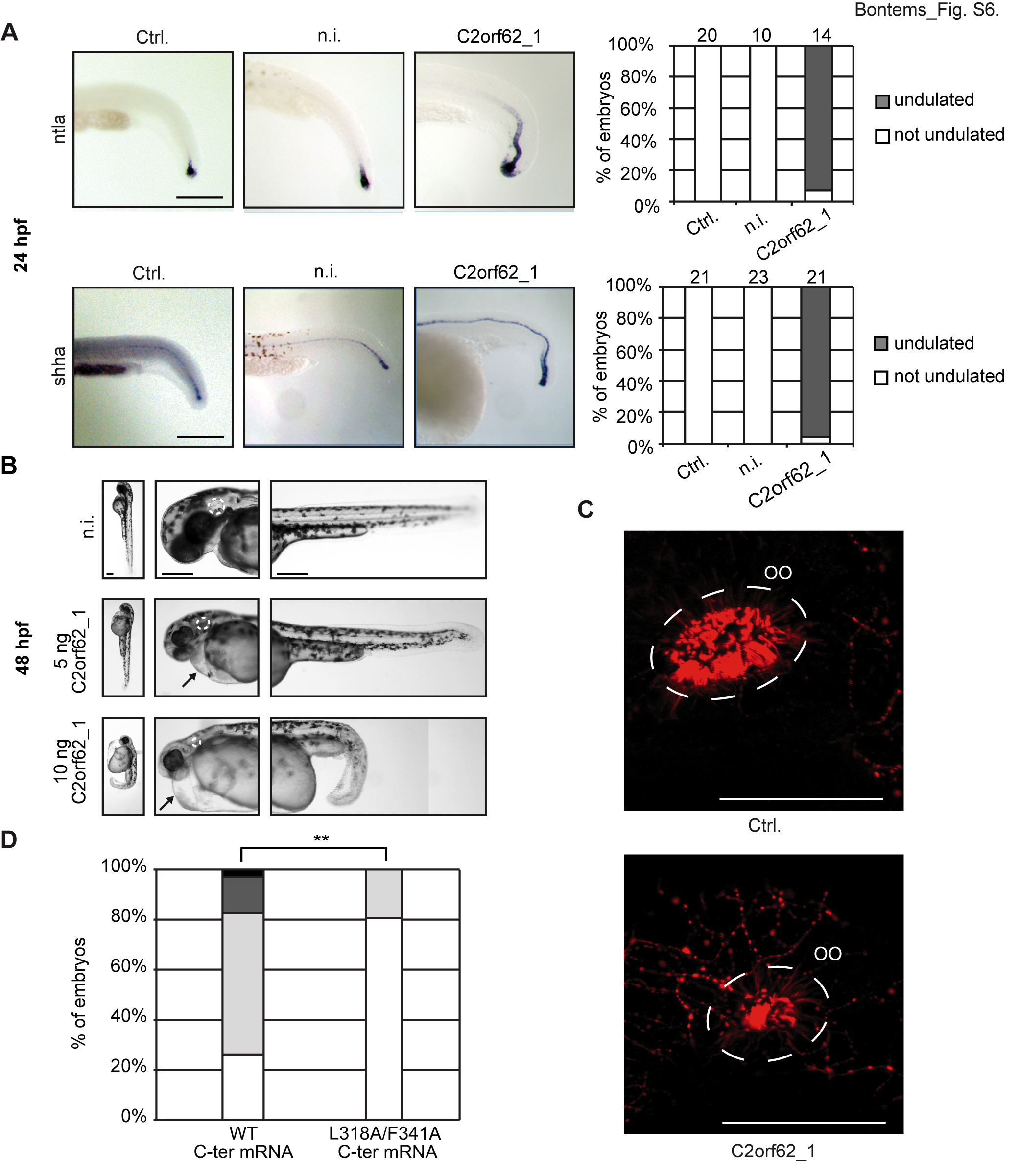

Supplement: Figure S6 — (related to Fig. 6 ). Knockdown of zC2orf62 results in notochord undulation and ciliogenesis defects in the olfactory organ. (A) In situ hybridization of ntla and shha at 24 hpf shows that more than 90% of C2orf62_1 MO-injected embryos have an undulated notochord, in contrast to controls (lateral views, scale bars, 200 µm). (B) Embryos were either not injected (n.i.) or injected with 5 or 10 ng C2orf62_1 MO and observed at 48 hpf. C2orf62_1 MO-injected embryos display a curved body, heart edema (arrow), and a small head with incorrectly defined brain structures, small eyes and small ears (dashed circle). Scale bars, 200 µm. (C) Embryos were injected either with 2.5 ng Ctrl. MO or C2orf62_1 MO, and ciliated cells from the olfactory organ (OO) were visualized using anti α-acetylated tubulin at 48 hpf. The olfactory organs of C2orf62_1 MO-injected embryos are smaller and display less ciliated cells than controls. Scale bars, 50 µm. (D) Embryos were injected with 850 pg mRNA coding for the C-terminal part (aa 276-356) of zC2orf62,either wild type (WT C-ter) or alanine-mutated on Leu-318 and Phe-341 (L318A/F341A C-ter), and observed 24 h later. Phenotypic traits were classified into four categories, each being associated with one grey code, as reported in Fig. 6A: normal (white, indistinguishable from controls), severe (clear grey, defect of brain structure formation and short body axis), very severe (dark grey, indistinguishable left/right or front/back polarity), and lethal (black). The bar graph shows the percentage of embryos in each category. The percentage of normal (white) embryos is significantly higher for L318A/F341A C-ter mRNA-injected embryos than for WT C-ter mRNA-injected embryos (25.7% for WT C-ter mRNA and 80.6% for L318A/F341A C-ter mRNA P = 0.0078, Student's t test). (TIF) [file pone.0086476.s006.tif]
