# Supplementary material for: C2orf62 and TTC17 Are Involved in Actin Organization and Ciliogenesis in Zebrafish and Human
Source: PLoS One. 2014 Jan 27;9(1):e86476. doi: 10.1371/journal.pone.0086476 (PMC3903541; doi:10.1371/journal.pone.0086476)
Supplement: Table S1 — (related to Fig. 4 ). Yeast two-hybrid results. 10 positive clones obtained in the yeast two-hybrid screen were identified by sequencing. Clones labeled as “Not relevant” are typical yeast two-hybrid artefacts. (DOCX) [file pone.0086476.s007.docx]

**Table S1**

| ***UniProt AC*** | ***Gene name*** | ***Protein description*** | ***Start*** | ***Comments*** |
| --- | --- | --- | --- | --- |
| P05388 | RPLP0 | 60S acidic ribosomal protein P0 | 1 | Not relevant |
| Q9H8M2 | BRD9 | Bromodomain-containing protein 9 | 101 | Not relevant |
| **O75569** | **PRKRA** | **Interferon-inducible double stranded RNA-dependent protein kinase activator A** | **1** |  |
| **O75569** | **PRKRA** | **Interferon-inducible double stranded RNA-dependent protein kinase activator A** | **1** |  |
| P05412 | JUN | JUN protein | 247 | Not relevant |
| P19838 | NFKB1 | Nuclear factor NF-Kappa-B p105 subunit | 569 | Not relevant |
| Q8TEY7-2 | USP33 | Ubiquitin carboxyl-terminal hydrolase 33 | 655 | Not relevant |
| **Q8TEP8** | **CEP192** | **Centrosomal protein of 192 kDa** | **1501** | **C-terminus** |
| **Q96AE7** | **TTC17** | **Tetratricopeptide repeat protein 17** | **995** | **TPR4-6** |
| Q13435 | SF3B2 | Splicing factor 3B subunit 2 | 1 | Not relevant |
